# QupKake: Integrating Machine Learning and Quantum Chemistry for Micro-p*K*_a_ Predictions

**DOI:** 10.1021/acs.jctc.4c00328

**Published:** 2024-06-04

**Authors:** Omri D. Abarbanel, Geoffrey R. Hutchison

**Affiliations:** † Department of Chemistry, University of Pittsburgh, 219 Parkman Avenue, Pittsburgh, Pennsylvania 15260, United States; ‡ Department of Chemical and Petroleum Engineering, University of Pittsburgh, 3700 O’Hara Street, Pittsburgh, Pennsylvania 15261, United States

## Abstract

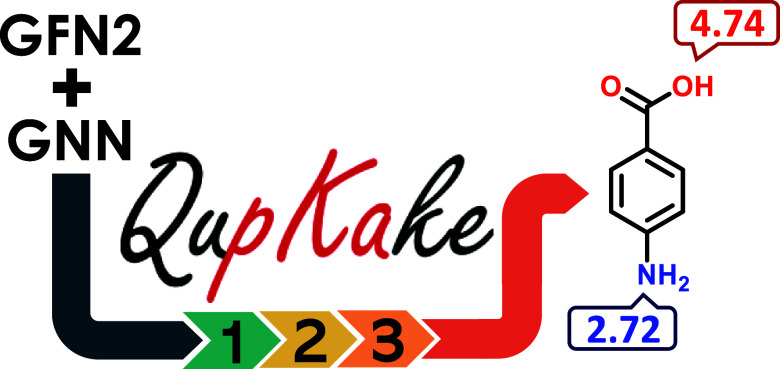

Accurate prediction of micro-p*K*_a_ values is crucial for understanding and modulating the acidity and basicity of organic molecules, with applications in drug discovery, materials science, and environmental chemistry. This work introduces QupKake, a novel method that combines graph neural network models with semiempirical quantum mechanical (QM) features to achieve exceptional accuracy and generalization in micro-p*K*_a_ prediction. QupKake outperforms state-of-the-art models on a variety of benchmark data sets, with root-mean-square errors between 0.5 and 0.8 p*K*_a_ units on five external test sets. Feature importance analysis reveals the crucial role of QM features in both the reaction site enumeration and micro-p*K*_a_ prediction models. QupKake represents a significant advancement in micro-p*K*_a_ prediction, offering a powerful tool for various applications in chemistry and beyond.

## 1 Introduction

The acid–base dissociation constant (p*K*_a_) is a fundamental physicochemical property of molecules, with broad applications in organic synthesis, environmental chemistry, medicinal chemistry, and drug design and discovery.^1,2^ The p*K*_a_ value of a molecule reflects its relative propensity to donate or accept a proton and can have a significant impact on its solubility, membrane permeability, protein binding affinity, stability, and other properties critical to drug development.^1−3^

For polyprotic acids and bases, it is essential to consider the micro-p*K*_a_ values, which is the term of art for microscopic or microstate p*K*_a_, of individual protonation and deprotonation sites. Micro-p*K*_a_ values refer to the p*K*_a_ values of specific sites on a molecule, rather than the overall p*K*_a_ value of the entire molecule. Knowing the micro-p*K*_a_ values of a polyprotic molecule can help us understand its behavior at different pH levels and design drugs or other molecules with optimal properties.

The chemical space of small, “drug-like” molecules is vast, estimated to be approximately 10^60^.^4^ Experimental p*K*_a_ evaluation of all potential molecules is impractical, as only a modest number of reliable experimental p*K*_a_ values are available. As a result, researchers have developed various computational approaches to predict p*K*_a_ values, broadly classified into two categories: quantum mechanical (QM) and machine learning (ML) models.

QM models use different computational methods, such as density functional theory (DFT), semiempirical methods, or quantum mechanics/molecular mechanics (QM/MM) to compute the thermodynamics of protonation or deprotonation and thus the acid–base equilibrium. These methods are based on the principles of quantum mechanics and can provide accurate predictions of p*K*_a_ values, with root-mean-square errors (RMSEs) ranging from 0.6 to 1.6 p*K*_a_ units, particularly for related species, but they are computationally expensive.^5−10^

ML models instead use ML algorithms, such as random forests and graph neural networks (GNNs), to predict p*K*_a_ values, based on training on either previously computed or experimental data. ML models are less computationally expensive to evaluate than QM models, but they are generally not as accurate on novel compounds with RMSEs ranging from 0.7 to 1.5 p*K*_a_ units. However, they are still useful for screening large numbers of molecules to identify potential drug candidates.^11−16^

There are several pitfalls in designing new micro-p*K*_a_ models. The first one is to consider the correct tautomer, most likely to be the abundant form of the molecule in question at neutral pH in aqueous solution. This can lead to incorrect assignment of reaction sites in both the training and prediction steps of ML model development. The second is the need to consider the molecule as a whole, including electronic and steric effects which may modulate the reactivity of functional groups and individual sites. The third is the lack of publicly available high-quality experimental p*K*_a_ data sets with enough data points to ensure generalization of the ML models and avoid overfitting. Although some models use private or commercial data sets,^12,13,16^ a different approach, such as transfer learning,^17^ is needed to overcome this challenge, until more experimental p*K*_a_ measurements become available.

In this work, we introduce a new method, QupKake (**Qu**antum **p*K***_**a**_ graph-neural-networ**k e**stimator), a model which combines GNNs with QM features from the GFN2-xTB semiempirical method,^18^ for the prediction of small-molecule micro-p*K*_a_. By combining both QM and ML, we are able to achieve state-of-the-art micro-p*K*_a_ prediction accuracies, with RMSEs between 0.5 and 0.8 p*K*_a_ units on experimental test sets, significantly lower than previous models. Additionally, QupKake is open-source with the intention of helping advance scientific research and discovery. The complete source code and all the data sets used in the training and testing of QupKake can be found on GitHub at https://github.com/hutchisonlab/QupKake.

To achieve these results, we applied several techniques and methodologies. A three-step workflow includes a QM-based tautomer search step with an implicit water solvation model, enumeration of reaction sites using QM knowledge and GNNs, and a GNN-based micro-p*K*_a_ prediction model that was trained using transfer learning. QupKake’s unique design shows that combining the best of both worlds, QM and ML, can lead to better models and open up new possibilities for molecular design.

## 2 Methods

**Figure 1:**
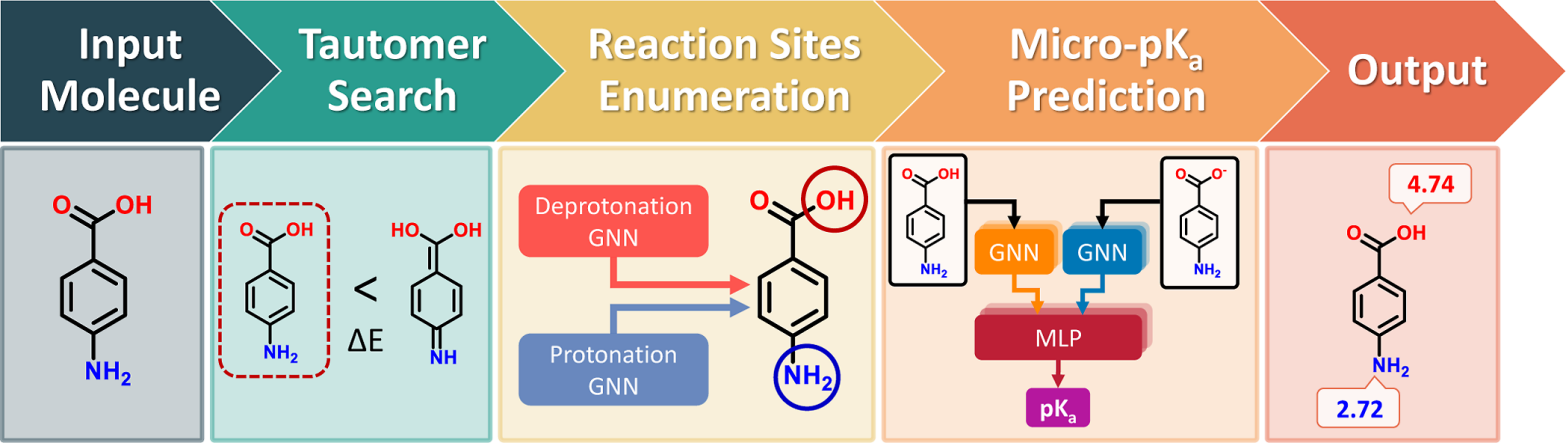
QupKake’s workflow. The input molecule goes through three steps: tautomer search, reaction site enumeration, and micro-p*K*_a_ prediction. The output is the micro-p*K*_a_ value of each reaction site.

The workflow of QupKake has three main parts: tautomer search, reaction site enumeration, and micro-p*K*_a_ prediction (Figure 1). These steps are designed to ensure the accuracy of the predicted micro-p*K*_a_ values.

- Tautomer search: QupKake identifies the most stable tautomer of the input molecule. This is important because the micro-p*K*_a_ value of a site can vary depending on the tautomeric form.
- Reaction site enumeration: QupKake enumerates all of the possible protonation and deprotonation sites on the molecule. This takes into account the chemical structure of the molecule and the protonation states of its neighbors.
- Micro-p*K*_a_ prediction: QupKake predicts the micro-p*K*_a_ values of the enumerated reaction sites.

### 2.1 Tautomer Search

Certain molecules undergo tautomeric transitions, where they can exist in different forms due to the movement of protons within their structure. This proton shuffle is influenced by factors like the solvent and typically leads to the dominance of one tautomer in a solution. These distinct tautomeric forms exhibit varying bonding arrangements, resulting in potentially different micro-p*K*_a_ values. Therefore, to understand the chemical and physical properties, it is essential to determine the most stable and prevalent tautomeric species within the solution.^19−21^

Although some ML models incorporate a tautomer search step, such as Schrödinger’s Epik,^13^ most do not.^8,10,12,14−16^ The identity of the most stable tautomer is a key factor for p*K*_a_ prediction, so incorporating a tautomer search step in an ML model should hypothetically yield more accurate results. Therefore, we employ the GFN2-xTB method to quickly identify the most stable tautomer.

The list of tautomers for each molecule was generated using the tautomer enumeration function of version 2022.03.3 of the RDKit software package.^22^ For simplicity of the workflow, the tautomer search focused on neutral compounds. The total electronic energy of each tautomer was then calculated using version 4.6.1 of the GFN2-xTB method^18^ with the analytical linearized Poisson–Boltzmann implicit solvation model in water.^23^ The lowest-energy tautomer, i.e., the most stable tautomer, was saved and used in the next steps, while the less stable tautomers were discarded. Consideration of tautomeric forms for acids, bases, and zwitterions is recognized as an area for future work, since the most stable tautomer of the neutral species may not be the most stable for other states.

### 2.2 Reaction Site Enumeration

In the next stage of the QupKake workflow, we focus on enumerating potential reaction sites, specifically atoms with a higher probability of either gaining or losing a proton within the most stable tautomeric form identified in the previous step. Many of the currently available micro-p*K*_a_ models rely on predefined SMARTS patterns^24^ to identify common acidic and basic groups. These patterns, however, can fail to consider other important molecular characteristics, such as the impact of neighboring groups or electrostatics on the reactivity of the reaction site. Furthermore, the use of different sets of SMARTS patterns by various models can lead to inconsistencies in the identification of reaction sites.

For this study, we used a tool called Conformer-Rotamer Ensemble Sampling Tool (CREST),^25^ which employs GFN2-xTB calculations and has a protonation and deprotonation site screening function.^26^ For protonation site screening, CREST identifies lone pairs and π orbitals as possible protonation sites, followed by geometry optimization and the ranking of protomers by their total GFN2-xTB energies. For the deprotonation site screening, CREST iteratively removes protons from the input molecules, followed by geometry optimization and the ranking of protomers by their total GFN2-xTB energies.

We used the Protonation and Deprotonation Site Search option of CREST version 2.12^25^ together with GFN2-xTB version 4.6.1. Because CREST tests all possible structures, it finds sites that are chemically unreasonable or rarely occurring, such as the protonation of aromatic carbons. This behavior has been reported previously and is attributed to a low activation barrier for proton-transfer reactions predicted by GFN2-xTB, compared to other methods.^18,27^ Therefore, we constrained CREST to only output structures up to 10 eV higher in energy than the lowest-energy structure. This reduces the number of possible protonation or deprotonation sites found by CREST to only the most stable reaction sites.

Our data set consists of 1,475,879 molecules extracted from version 32 of the ChEMBL online database.^28,29^ The molecules were filtered to include only organic molecules (atoms H, C, N, O, S, P, F, Cl, Br, and I) with ChemAxon^30^ acidic or basic p*K*_a_ values between 0 and 14. In this work, acidic p*K*_a_ refers to the p*K*_a_ of a removal of an acidic proton, while basic p*K*_a_ refers to the protonation of a base. To further augment the data set and teach the model about enantiomers, we converted each chiral molecule in the ChEMBL data set to its enantiomer by inverting all chiral centers in the SMILES representation, recognizing that enantiomers intrinsically have the same p*K*_a_ values and features. This yielded an additional 376,202 molecules, for a total of 1,852,081 molecules. Molecular descriptor distributions of the data set are shown in Figure S1 in the Supporting Information.

CREST protonation and deprotonation was then performed on each molecule in the data set. CREST output structures were compared with the input molecule using openbabel version 3.1.1^31^ in order to identify reaction sites. This was done separately for the protonation and deprotonation processes to generate a separate data set for each reaction. Since CREST performs reactive molecular dynamics, molecular rearrangement or degradation can occur after protonation or deprotonation, such as ring closure or splitting. Therefore, any molecule with a structural change other than an addition or removal of a proton was removed from the data set. In total, there are 1,331,870 protonated molecules with a total of 2,265,676 protonation sites and 1,214,117 deprotonated molecules with a total of 1,799,457 deprotonation sites.

Comparison of the protonation and deprotonation sites found by CREST versus those identified using SMARTS patterns is intended to provide insights into their agreement and to explore whether CREST identifies reaction sites beyond those captured by SMARTS patterns and vice versa. For this purpose, we compared the reaction sites in our data set with the SMARTS patterns compiled by Pan et al.,^16^ encompassing 54 acidic group patterns and 89 basic group patterns. This comparison with SMARTS patterns from previous literature is not to consider them as a “gold standard” but rather to evaluate how CREST’s findings align with established patterns in the literature and to highlight any novel insights CREST might provide.

Overall, CREST and the SMARTS patterns agreed on 53.08% of the protonation sites and 72.32% of the deprotonation sites. This indicates that CREST results and the SMARTS patterns generally agree on the location of protonation and deprotonation sites. However, there are also some significant differences.

For protonation, 40.67% of the sites were found using the SMARTS patterns but not with CREST, while 6.24% of the sites were found with CREST but not with the SMARTS patterns. This discrepancy underlines that while CREST may identify some unique protonation sites not captured by the SMARTS patterns, due to its QM calculations that consider nonlocal and nonbonding interactions, the SMARTS patterns, on the other hand, tend to identify a broader array of protonation sites without regard to the entire molecule or relative energies. This results in a significant number of potential sites, including some higher in energy and thus a decreased agreement between the two methods. For deprotonation, the percentages are slightly closer, with 24.67% of the sites found with the SMARTS patterns but not with CREST and 3.31% found with CREST but not with the SMARTS patterns. This suggests that CREST and the SMARTS patterns are more similar in their identification of deprotonation sites, but the SMARTS patterns tend to find more possible deprotonation sites. This comparison does not expect a complete concurrence between SMARTS patterns and CREST, particularly given the limitations of SMARTS in capturing complex QM interactions. While deriving tautomerization rules from quantum chemical calculations has been performed successfully,^20,21^ as of yet, we have not observed the same for protonation or deprotonation rules. We hope that this work and data set can help to provide a basis for similar efforts.

Overall, a comparison of the CREST results and the SMARTS patterns reveals that they generally agree on the location of protonation and deprotonation sites, albeit with differences. Several examples of the different predictions of protonation and deprotonation sites are shown in Figures S8 and S9 in the Supporting Information, respectively.

Again, while the SMARTS patterns may be useful, we focus on CREST reaction sites because it uses GFN2-xTB calculations to rank the relative thermodynamics of protonated and deprotonated molecules by total electronic energy, which will be useful later. However, its exhaustive approach involving meta-dynamics and molecular dynamics simulations contributes to its relatively slow performance, taking up to several hours for each molecule. Therefore, an ML model trained on the CREST data set can be used as a surrogate for CREST reaction site determination to provide fast, high-accuracy predictions.

#### 2.2.1 GNN Models

ML methods have been successfully applied to predict molecular properties in recent years,^32−40^ including macro- and micro-p*K*_a_.^10,12,13,15,16^ GNNs are a type of ML model that can be used to learn representations of graphs, where nodes represent atoms and edges represent bonds. GNNs have been shown to be effective for molecular property prediction tasks.^11,13,16,41−46^ In this study, we constructed and trained two GNNs on the CREST data set to predict protonation and deprotonation sites. We found that our GNN models outperformed CREST in terms of prediction accuracy and speed. Our results suggest that GNNs can be used to accelerate QupKake’s workflow by identifying potential reaction sites more efficiently.

We generate a molecular graph representation of each molecule using version 2.3.0 of the PyTorch Geometric^47^ package. For each atom (node), bond (edge), and molecule (graph), we generated a set of features using RDKit and GFN2-xTB. For a complete list of features, see Table S1 in the Supporting Information. Our protonation and deprotonation site prediction models are node-level prediction models. The input to each model is a graph representation of a molecule in the shape of (61, *N*), where *N* is the number of atoms. The output of each model is a one-dimensional binary vector in the shape of (1, *N*), where each element of the vector indicates whether the corresponding atom is a predicted protonation or deprotonation site, respectively.

We split the CREST data set into train, validation, and test sets in an 80:10:10 ratio, respectively. We trained the models using Python version 3.9, PyTorch^48^ version 2.0.0, and PyTorch Lightning^49^ version 2.0.2. We tested three different GNN architectures: Graph Convolution Network,^50^ Graph Attention Network,^51^ and Graph Transformer Network.^52^ We used Optuna^53^ version 3.2.0 to find the best architecture and hyperparameters for the models, with the goal of maximizing accuracy. Early stopping of the model training was used to prevent overfitting.

To confirm that the GFN2-xTB features contribute significantly to the models’ performance, we used the Integrated Gradients algorithm,^54^ as implemented in version 0.6.0 of the Captum Python package,^55^ to quantify the importance of each feature in predicting the reaction site. The Integrated Gradients algorithm is a model-agnostic attribution method that can be used to explain the predictions of any ML model. It works by computing the gradient of the model’s output with respect to its input features and then integrating the gradients over a path from a baseline input to the actual input. The resulting values represent the importance of each feature in contributing to the prediction of the model.

### 2.3 Micro-p*K*_a_ Prediction Model

**Figure 2:**
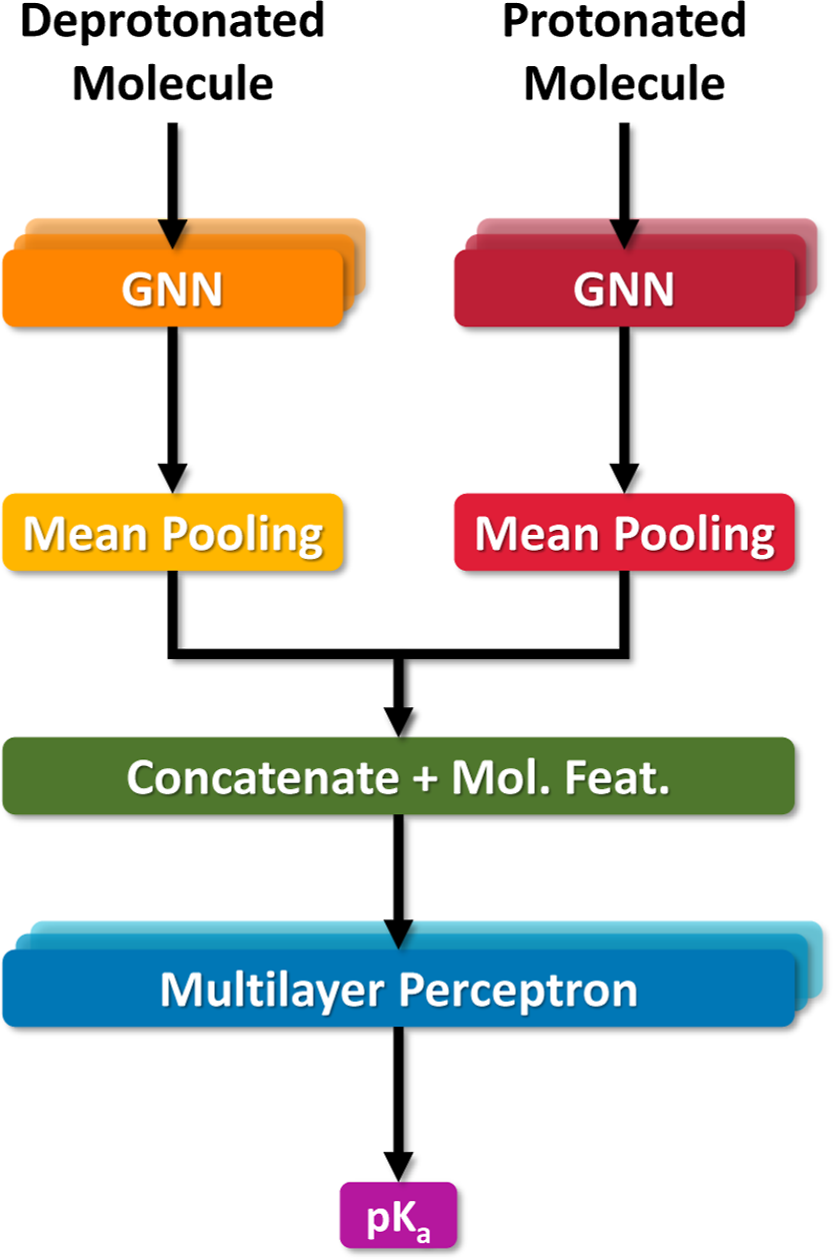
Simplified micro-p*K*_a_ model architecture. The model takes in two input molecules, where one molecule is the protonated version of the other. The model’s output is the p*K*_a_ value of the protonation/deprotonation reaction between the two species. See Figure S13 in the Supporting Information for the full model architecture.

The final step in QupKake’s workflow is the prediction of the micro-p*K*_a_ values for the reaction sites previously predicted. The model architecture follows a similar schematic to the one proposed by Mayr et al.,^14^ in which the input consists of a graph representation of both the original input molecule and its protonated or deprotonated version, depending on the reaction site. Each input molecule undergoes several GNN layers, followed by a global mean graph pooling layer. The outputs are then concatenated, together with the molecular features vector of each input molecule, into a one-dimensional vector. This vector is passed to several linear layers that outputs the p*K*_a_ of that site (Figure 2). The implementation of this model used the same tools and packages as described above.

Comprehensive and high-quality public micro-p*K*_a_ data sets that would provide enough diverse experimental values to train a model are hard to come by. While the iBond database^56^ contains over 30,000 equilibrium p*K*_a_ values in aqueous and nonaqueous solvents, it is not readily accessible and encompasses p*K*_a_ values across 46 different solvents, complicating its direct application for model training due to solvent diversity. Some models, such as Schrödinger’s Epik,^13^ use proprietary data that are not accessible to others, inhibiting the progress of scientific discovery. Baltruschat and Czodrowski have curated a data set of ∼6000 organic compounds with experimental p*K*_a_ values^15^ and used ChemAxon’s Marvin^30^ to find the reaction site. Molecular descriptors and the distribution of p*K*_a_ values for the experimental data set can be found in Figures S3 and S4 in the Supporting Information. However, 6000 experimental data points are not enough to adequately train a GNN model.^57^ Furthermore, during model development, we have found that the experimental data set requires augmentation and cleaning as some molecules had to be neutralized, had a chemically improbable assignment of the reaction site, or had calculation errors with GFN2-xTB. This narrowed the experimental data set to 5637 compounds.

To mitigate this problem, the model was trained on the previously mentioned ChEMBL data set, consisting of ∼2.5 million predicted acidic and basic p*K*_a_ values over ∼1.5 million molecules. As the molecules in this data set have been previously processed by CREST, the lowest-energy protomer or deprotomer was assigned to the predicted p*K*_a_ value. That is, Marvin’s most basic p*K*_a_ prediction was assumed to describe the protonation reaction of the molecule and the most stable protomer found by CREST. The same applies to the most acidic p*K*_a_ and the molecule’s most stable deprotomer. The assignment of a predicted macro-p*K*_a_ value to a reaction center and treating it as a micro-p*K*_a_ value is because in most cases, CREST found only one acidic or basic reaction center that was energetically accessible per molecule, which is in agreement with our assumption. In cases where there were multiple reaction centers, this assumption can be flawed, but it is how we were able to achieve a diverse training set. As before, the model was divided into training, validation, and testing sets in an 80:10:10 ratio, respectively.

Transfer learning was then used to fine-tune the model with the 5637 experimental micro-p*K*_a_ values, which was randomly split to an 80:20 ratio of training and validation sets. Transfer learning has been a widely used technique in the ML world for fine-tuning a pretrained model on new information with the purpose of increasing accuracy or improving performance on another related task.^58−60^ In this case, the pretrained model was able to take advantage of its existing knowledge of molecular structure and properties to learn to predict micro-p*K*_a_ values more accurately.

To test the performance of the model and compare it to other available models, we used two public data sets also curated by Baltruschat and Czodrowski.^15^ Those data sets consist of 279 molecules from the Novartis drug company, and the other data set consists of 123 molecules with experimental p*K*_a_ values from different literature sources. Molecular descriptors and the distribution of p*K*_a_ values for the experimental data set can be found in Figures S5 and S6 in the Supporting Information. The model’s performance was also compared with the data sets of statistical assessment of protein and ligand modeling (SAMPL) in public challenges of prediction SAMPL6,^61^ SAMPL7,^62^ and SAMPL8^63^ micro-p*K*_a_ prediction public challenges.

## 3 Results and Discussion

### 3.1 Reaction Site Enumeration

#### 3.1.1 Model Performance

The tuned and trained micro-p*K*_a_ prediction models were evaluated on an out-of-sample set of 133,188 molecules with protonation sites and 121,413 molecules with deprotonation sites, randomly selected from the CREST data sets. The models achieved very high accuracy, with 98.2 and 98.8% accuracy for protonation and deprotonation site enumeration, respectively. These results are shown in Figure S10. The optimized hyperparameters are listed in Table S2 in the Supporting Information.

#### 3.1.2 Feature Importance

To better understand which features are the most important for the protonation site enumeration model, we performed atom and bond feature importance analysis as described in Section 2.2.1 (Figure S11). The most important atom feature is “Atom Type: N”, which indicates whether the atom is a nitrogen atom. This makes chemical sense, as nitrogens are more likely to be protonated than other types of atoms in neutral molecules. Furthermore, 96% of the protonated atoms in the CREST data set are nitrogens (Figure S7), which supports the importance of this feature.

The next two most important atom features are the covalent coordination number (“xTB Coord Number”) and the susceptibility to radical attack Fukui(0) index [“xTB Fukui(0)”], both of which are calculated using GFN2-xTB. This shows that QM features contribute significantly to the model’s performance.

The most important bond feature is “Bond Type: SINGLE”, followed by the “Wiberg Bond Order”, which is calculated from the GFN2-xTB results. The computed bond order is a continuous real value based on electron density and can thus indicate variations in bond strength.^64^ This corroborates the importance of QM features, as well as the importance of single bonds in protonation reactions.

Overall, feature importance analysis shows that the model is able to leverage both topological and QM features to accurately predict protonation sites. This is an important finding as it demonstrates that the model can be used to predict protonation sites for a wide range of molecules, even those for which experimental data is not available.

Similarly, to better understand which features are the most important for the deprotonation site enumeration model, we performed atom and bond feature importance analysis (Figures S12).

The most important atom feature is “Is HBD”, which indicates whether the atom is a hydrogen-bond donor. This makes chemical sense, as hydrogen-bond donors are more likely to be deprotonated than other types of atoms. The highest GFN2-xTB feature, the atom’s partial charge (“xTB Partial Charge”), is only in seventh place, indicating that QM features are less important for deprotonation site prediction than for protonation site prediction.

Similar to the protonation site enumeration model, the most important bond feature is “Bond Type: SINGLE”. However, the GFN2-xTB calculation “Wiberg Bond Order” feature is only the fifth most important feature, again indicating that QM features are not as important for deprotonation site prediction as for protonation site prediction.

Overall, feature importance analysis shows that the deprotonation site enumeration model is less reliant on QM features than the protonation site enumeration model.

Despite the lower importance of QM features for deprotonation site prediction, the model is still able to achieve high accuracy. This is likely because the model is able to learn complex relationships between the topological and chemical properties of the molecule.

### 3.2 Micro-p*K*_a_ Prediction Model

#### 3.2.1 Model Performance

**Figure 3:**
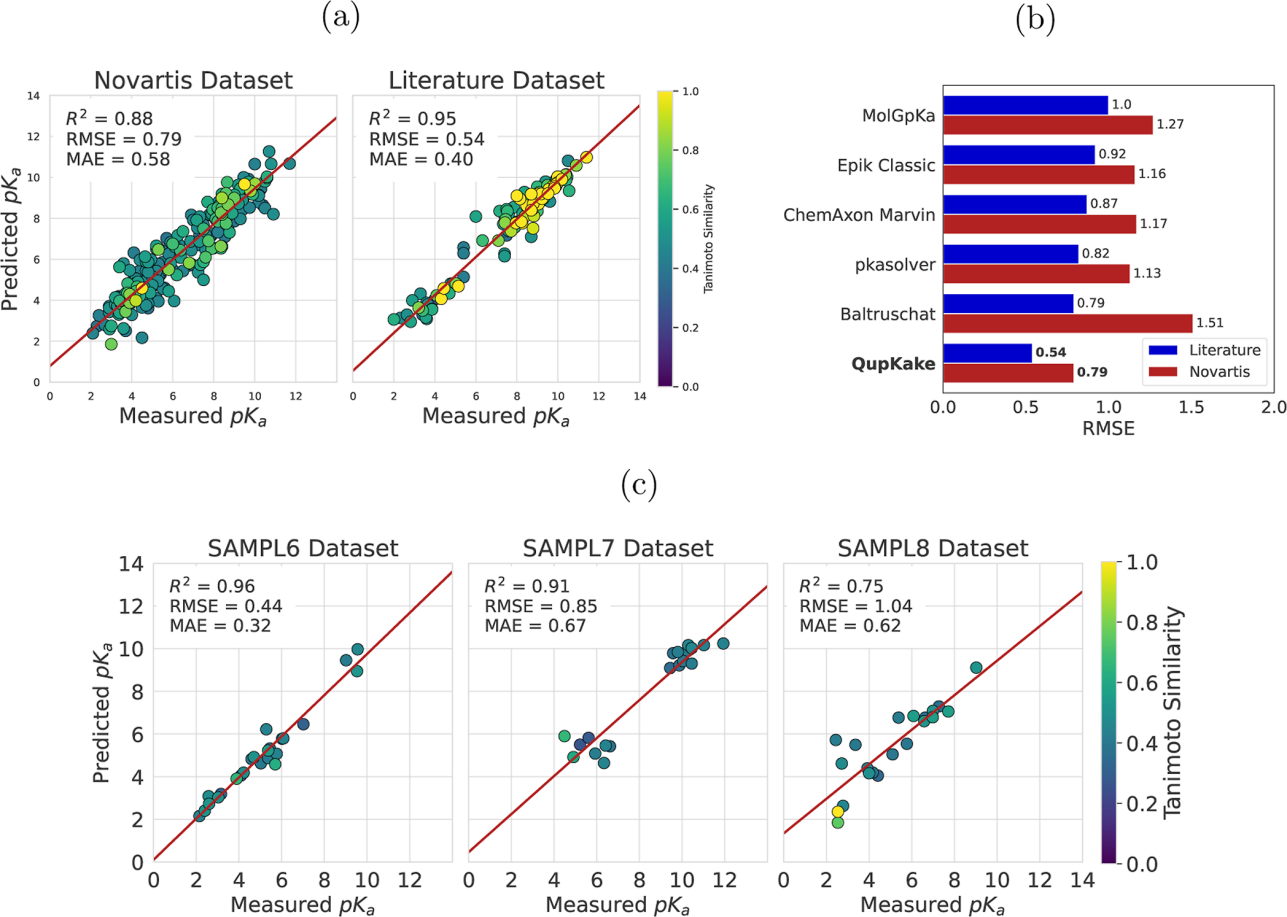
Micro-p*K*_a_ predictions versus the measured micro-p*K*_a_ values of the (a) Novartis data set and Literature data set, as well as the (c) SAMPL6, SAMPL7, and SAMPL8 data sets. Data points are colored according to the highest Tanimoto similarity score of the molecule in the test set versus the molecules in the experimental training set. The best-fit linear regression line is shown in red. (b) RMSE comparison of the Novartis and Literature data sets between QupKake and five other models. The RMSE values for the five other models were obtained from Mayr et al.^14^

The tuned and trained micro-p*K*_a_ prediction model was validated on five external test sets: the Novartis data set (containing 280 molecules), the Literature data set (containing 122 molecules), and the SAMPL6, SAMPL7, and SAMPL8 data sets (containing 24, 20, and 21 molecules, respectively). The results of this evaluation are shown in Figure 3a,c. The optimized hyperparameters are listed in Table S3 in the Supporting Information.

On the Novartis and Literature test sets, the model achieved low prediction errors with RMSEs of 0.79 and 0.54 p*K*_a_ units, respectively, and mean absolute errors (MAEs) of 0.55 and 0.39 p*K*_a_ units, respectively. The model also achieved high coefficients of determination (*R*^2^) for both test sets, with values of 0.88 and 0.95, respectively. These results demonstrate that the model is able to accurately predict micro-p*K*_a_ values for a wide range of organic molecules.

To put QupKake’s high performance into context, Figure 3b shows a comparison of the RMSE of QupKake versus five other models on the Novartis and Literature data sets. Those models include MolGpKa,^16^ Schrödinger’s Epik Classic,^65^ ChemAxon’s Marvin,^30^ pkasolver,^14^ and the Baltruschat model from the Czodrowski group.^15^ The RMSE values were obtained from Mayr et al.^14^ It is clear to see that QupKake significantly outperforms all other models, with RMSE differences of 0.34 and 0.25 p*K*_a_ units on the Novartis and Literature test sets between QupKake and the next-best models.

The Novartis and Literature test sets were both obtained from Baltruschat and Czodrowski,^15^ which stated that the test sets and the experimental training set do not have the same molecules, which could lead to the model “memorizing” values instead of learning. However, we have found that this is not the case, since several molecules appear in both these test sets and the experimental training set. To see if the model actually memorized the molecules and their p*K*_a_ values, or whether QupKake was able to generalize, we removed any molecules from the test sets with high Tanimoto similarity scores (<0.8) and compared the performance of the model with the rest of the molecules (Figure S15). Tanimoto similarity scores are defined as the ratio of the intersection of the two sets of fingerprints over the union of the two sets and have been widely used to calculate molecular similarities in various applications.^66^ The *R*^2^, RMSE, and MAE of the Novartis data set remained the same, while the RMSE and MAE of the Literature data set slightly increased to 0.59 and 0.43, respectively, while *R*^2^ was unchanged. These results show that the model did not simply memorize values and was able to learn and generalize on a range of different species and p*K*_a_ values. Furthermore, we have not found any correlation between the Tanimoto similarity score and the p*K*_a_ error (Figure S14), again suggesting that the QupKake model has strong generalization.

To illustrate the benefits of transfer learning in refining the model’s accuracy, we conducted experiments with two distinct models without transfer learning. The first is the initial model, trained solely on the ChEMBL data set without fine-tuning using the experimental data, which exhibited an increase in the RMSE for the Novartis and Literature test sets to 1.09 and 0.86, respectively, compared to the fine-tuned model (Figure S17). The second model, trained exclusively on the experimental data with identical hyperparameters to those of the fine-tuned model, demonstrated a more pronounced increase in RMSE for the Novartis and Literature test sets, reaching 1.79 and 1.31, respectively (Figure S18). These results affirm our hypothesis that transfer learning markedly improves the model’s performance.

Despite being trained on the ChemAxon data set, QupKake outperforms the Marvin program in identifying reaction sites that correlate more closely with experimental p*K*_a_ values in the Novartis and Literature data sets. This indicates that QupKake’s ability to identify accurate reaction sites generalizes well beyond the training data. Even when evaluated using the reaction sites identified by Marvin, QupKake still surpasses the performance of other micro-p*K*_a_ prediction models. On the Novartis and Literature test sets, QupKake achieves RMSEs of 1.00 and 0.59 p*K*_a_ units, respectively (Figure S16). These RMSEs are higher than those obtained using QupKake’s own reaction sites, but they remain lower than those of the other five models compared in Figure 3b. This demonstrates QupKake’s robustness and ability to provide accurate micro-p*K*_a_ predictions even when using reaction sites identified by external tools.

The model was also tested on the SAMPL6, SAMPL7, and SAMPL8 p*K*_a_ prediction challenges (Figure 3c). QupKake outperformed all of the submitted models for the SAMPL6^61,67^ and SAMPL8 models^63^ and would have been ranked first if the challenges were still open for submission. Table 1 shows the superior performance of QupKake on the SAMPL6 and SAMPL8 data sets compared to the best-performing submissions, as well as the latest Epik 7 Ensemble model from Schrödinger.^13^ QupKake performs slightly worse on the SAMPL7 data set and would be ranked second by RMSE compared to the other submissions.^62^

**Table 1:** Comparison of QupKake’s Accuracy versus the Top Ranked Submissions^67^ in the SAMPL6,^61,67^ SAMPL7,^62^ and SAMPL8^63^ p*K*_a_ Prediction Challenge^a^

SAMPL6				SAMPL7				SAMPL8			
model	RMSE	MAE	*R*^2^	model	RMSE	MAE	*R*^2^	model	RMSE	MAE	*R*^2^
QupKake	**0.44**	**0.32**	**0.96**	EC_RISM	0.72	0.53	0.93	QupKake	**1.04**	**0.62**	**0.75**
Epik 7 Ensemble	0.61	0.48	0.95	QupKake	**0.85**	**0.67**	**0.91**	DeeepGP	3.17	2.62	0.15
Grimme	0.68	0.58	0.94	IEFPCM/MST	1.82	1.30	0.56	3DS	3.44	2.49	0.27
S + p*K*_a_	0.73	0.59	0.93	DFT_M05-2X_SMD	2.90	2.28	0.03	ChemAxon	4.18	2.82	0.09
ACD/p*K*_a_ Classic	0.79	0.56	0.92	TZVP-QM	2.90	2.75	0.23	ECRISM	4.56	3.05	0.18
COSMOtherm p*K*_a_	0.90	0.71	0.90	Gaussian process	3.49	2.91	0.30	ZhiyiWu	4.73	3.37	0.05
MoKa	0.94	0.77	0.88	DFT_M06-2X_SMD	5.12	2.56	0.20	uESE_extra	6.80	5.33	0.09
Epik Classic	0.95	0.78	0.91	Gaussian-corrected	5.36	5.12	0.76				

a The table is sorted from lowest to highest RMSE of the models in each SAMPL challenge. While the Epik 7 Ensemble Model^13^ was not submitted to the SAMPL6 challenge, we included it here as it is the most recently published micro-p*K*_a_ prediction model, as well as for providing its performance on the SAMPL6 data set.

Beyond ranking the performance on test sets, the model should be evaluated for trends in the most accurate and least accurate micro-p*K*_a_ predictions to consider potential chemical motifs and model bias on certain acidic or basic groups. Figures S19 and S20 in the Supporting Information show the 20 most accurate and least accurate micro-p*K*_a_ predictions on the Novartis data set. No clear pattern is observed, suggesting that there is no noticeable bias of the model. Although there are several examples of poor predictions on the acidic p*K*_a_ of amides in Figure S20, it is not a significant trend across the entire testing set. The RMSE of only acidic amides in the Novartis data set is 0.94 p*K*_a_ units, which, while slightly higher than the RMSE of the entire set (0.79), does not indicate clear bias against these groups.

Additionally, Thapa and Raghavachari compiled a set of organic molecules categorized by the functional group. They calculated p*K*_a_ values using high-level QM methods, employing both implicit and explicit water solvation models. Their results were tabulated for each group.^68^ By applying the QupKake model to each functional group list, we can assess whether QupKake exhibits differential p*K*_a_ prediction accuracy across groups (Tables S4–S15). As anticipated, QupKake demonstrates higher performance on functional groups well-represented within the training set. These include nitrogen-containing aromatic heterocycles, primary and secondary amines, carboxylic acids, anilines, and benzoic acids. On the contrary, groups less prevalent in the training data, such as aliphatic alcohols and thiols, phenols, and thiophenols, yielded lower precision. Notably, “carbon acids” (i.e., deprotonation of aliphatic carbon atoms) were absent from the training set, making the current model unable to predict the reaction site for this group.

As with any ML model, the accuracy and precision of the model output is directly related to the accuracy and precision of the training data. As mentioned before, high-quality micro-p*K*_a_ data is hard to obtain. Due to that, QupKake is trained only on one p*K*_a_ value per compound of a relatively small section of the vast chemical space. Therefore, we acknowledge that we can only have high confidence in the most acidic or basic micro-p*K*_a_ values that QupKake predicts, while we have less confidence in the micro-p*K*_a_ predictions of additional reaction sites in compounds with multiple sites (e.g., polyprotic acids). Further experimental p*K*_a_ data, for example, from automated characterization, would greatly improve the accuracy of future models.

Overall, the results of the external evaluation demonstrate that the tuned and trained QupKake prediction model is a highly accurate and reliable tool for predicting micro-p*K*_a_ values for organic molecules. The model outperforms all other state-of-the-art models on a variety of benchmark data sets and is able to generalize to new data, including examples which are more challenging than the training data. This suggests that the model could be used to predict micro-p*K*_a_ values for a wide range of organic molecules, including drug candidates and other molecules of interest.

#### 3.2.2 Feature Importance

As with the reaction site enumeration models, feature importance analysis can give useful insights into which features contribute to the micro-p*K*_a_ prediction model, such as whether including QM features improves the model performance. We performed feature importance analysis on the atomic, bond, and molecular features using the Integrated Gradients algorithm as described earlier. However, as the micro-p*K*_a_ prediction model’s architecture (Figure S13) is more complex than the reaction site enumeration models, as well as being a graph regression model, compared to a node classification model in the case of the site enumeration models, feature importance analysis is less straightforward.

In the micro-p*K*_a_ model architecture, the atomic and bond feature vectors of each atom and bond in both the protonated and deprotonated molecules are first passed through several GNN layers, which are then pooled into a one-dimensional vector. It is then concatenated with the molecular feature vectors and passes through several linear layers, which output the predicted micro-p*K*_a_. As the feature vectors, especially the atomic and bond features, have gone through several transformations, it can be difficult to deduce how and why each feature affected the model’s performance.

Figure S21a shows the importance of the atomic features, with “Atom Type: N” having the highest score. We hypothesized that this is due to the abundance of nitrogen reaction sites found by CREST and therefore by the reaction site GNNs. The next important atomic feature is the “xTB Alpha”, which is the atomic polarizability calculated by GFN2-xTB. This indicates that QM features provide useful information to the model and improve its performance. The next features by importance score include “Is HBA”, “Formal Charge: 0”, and “Atom Type: O”, which, as before, might indicate their abundance in the data set.

Similarly, the most important bond features shown in Figure S21b are “Is In Ring” and “Is Conjugated”, indicating the high prevalence of aromatic rings in the data set. The “Wiberg Bond Order”, calculated by GFN2-xTB, is the third most important feature, again proving that QM features contribute to the model performance.

The molecular features did not pass through the GNN layers and thus should have a more direct and interpretable impact on the model. Figure S21c shows that the five RDKit features, “RadiusOfGyration”, “Eccentricity”, “Spherocity”, “FractionCSP3”, and “Asphericity”, have almost identical importance scores, while the protonation energy (“xTB-Energy”, the energy difference between the protonated and deprotonated molecules) has a very low score. This could indicate that there is a negligible correlation between GFN2-xTB energies and p*K*_a_s, while the RDKit features contribute similar information to the model. Improved semiempirical methods with better treatment of solvation effects^69^ or ML-based models^70^ may improve the influence of these molecular features.

### 3.3 Model Speed

Although GFN2-xTB is a relatively fast semiempirical method, especially compared to higher-level methods,^71^ it is significantly slower than using RDKit alone to calculate graph-level features. As with many things in life, however, there is an inverse relationship between speed and accuracy and the QM features prove to be important in both tautomer selection and the ML models.

To evaluate how fast it takes for a molecule to pass through QupKake’s workflow, we performed a benchmark test using a server running a 3.85 GHz AMD EPYC 9374F CPU with 32 cores with a shared memory framework. Although ML tasks generally run faster on GPU, the rate-limiting steps in QupKake are the GFN2-xTB calculations, which do not take advantage of GPU acceleration. However, GFN2-xTB can utilize parallel processing on multiple cores to increase its calculation speed. Additionally, to achieve even better performance, we utilized Python’s multiprocessing module to parallelize the data set’s preprocessing.

**Figure 4:**
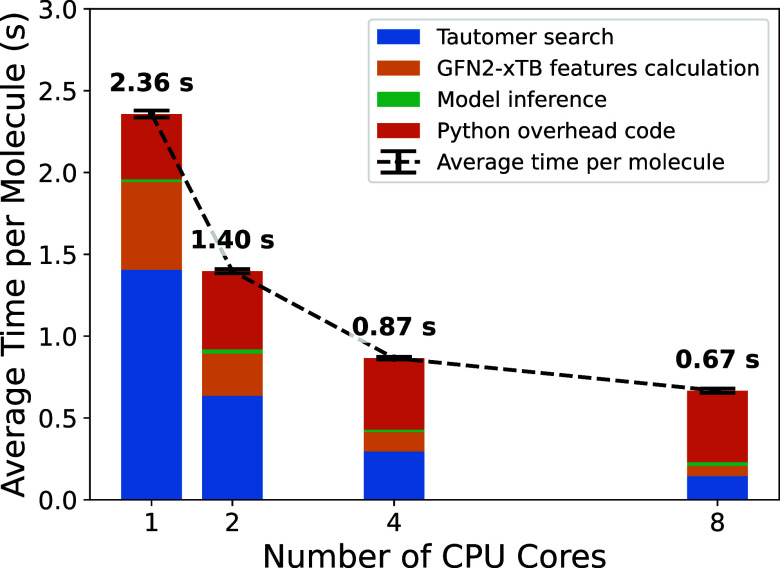
Average compute time per molecule across the 280 molecules in the Novartis test set as a function of the number of CPU cores, indicating time spent in the tautomer search calculations, GFN2-xTB feature calculations, ML model inference, and Python overhead from the QupKake model code, including RDKit descriptors. The error bars show the standard deviation of the average compute time per molecule over 10 trials.

Using the 280 molecules in the Novartis data set as our benchmark, we measured QupKake’s average compute time per molecule from start to finish, as well as how long each step took, as a function of the number of CPU cores (Figure 4). We repeated this 10 times to minimize random events that can skew the benchmark timings. The very small standard deviations, around 0.02 s per molecule in the single CPU core case, show that QupKake’s execution time is consistent. Of course, some variance is expected when different molecular sets are used.

It is clear that the tautomer search step, which uses GFN2-xTB to find the most stable tautomer, takes significant time to compute, followed by the calculations of the GFN2-xTB features for the reaction site search and the micro-p*K*_a_ prediction steps. As mentioned before, the actual model inference compute time is negligible, even when using a CPU.

As more CPU cores were used in parallel, the compute time for the tautomer search and the GFN2-xTB calculations decreased inversely at an approximately linear rate. That is, the average compute time for those steps using four CPU cores is approximately half the compute time using two cores and about a quarter compared to using a single core (Figure S22). These steps run well in parallel because multiple tautomers can be calculated at once, and many components of a quantum calculation such as GFN-xTB also run well in parallel.

In contrast, the compute time for the overhead Python code that could not be parallelized remained approximately the same regardless of how many CPU cores were used. Therefore, the overall speedup achievable by using more CPU cores is limited as it does not scale linearly with the number of cores (Figure S23). Thus, using more than two to four cores per molecule provides only a minor improvement in speedup.

In general, the use of multiple CPU cores can be a valuable tool to improve QupKake’s performance. However, the benefits of using multiple cores are not linear, and the overhead of using multiple cores can also be a factor. Therefore, in common cases such as the evaluation of multiple compounds in a set, it is more effective to run separate QupKake calculations in parallel rather than dedicating many cores to each.

## 4 Future Directions

We have shown that combining semiempirical QM features with ML improves the micro-p*K*_a_ predictions for small “drug-like” molecules. However, while GFN2-xTB is a relatively fast method, especially compared to higher-level methods,^71^ it is still slower than “pure” ML methods that use only RDKit features.^13,14,16^ As described above, the greatest bottlenecks in QupKake’s workflow are the GFN2-xTB calculations, and finding a faster replacement that provides similar or better accuracy can improve future versions of QupKake.

For example, the recently published MolTaut model^72^ which uses a GNN to rank tautomer stability in aqueous solutions could replace the current tautomer search step, which uses GFN2-xTB calculations to do so. Other models, such as Auto3D or AIMNet2,^70,73^ which uses a message-passing approach, can also be used to calculate the relative energies of the tautomers.

ML models can also be used to predict certain atomic and bond features, which can make the use of GFN2-xTB calculations obsolete. Features such as atomic partial charges,^74,75^ Fukui indices,^76^ and bond orders^77^ already exist and can be integrated with future iterations of QupKake. Other GFN2-xTB features that prove important for micro-p*K*_a_ predictions, such as atomic polarizabilities and coordination numbers, currently do not have published models. However, it is possible to build surrogate models to predict these values, in a manner similar to QupKake’s reaction site models, training on the calculated values from GFN2-xTB or a higher-level method.

Caldeweyher et al. recently introduced an alternative method for calculating various QM features, including coordination numbers, atomic polarizabilities, and partial charges.^78^ Their work presents kallisto, a program that employs equations parameterized to GFN2-xTB data to compute these features. While corrections to GFN2-xTB atomic polarizabilities have been noted,^79^ this approach offers the potential to significantly accelerate feature calculation to the level of GFN2-xTB computational efficiency, without directly utilizing the GFN2-xTB method.

## 5 Conclusions

In this work, we have presented QupKake, a novel and effective workflow for predicting micro-p*K*_a_ values of small organic molecules. QupKake leverages the power of GNNs and semiempirical QM features, namely, the GFN2-xTB method, to achieve exceptional accuracy and generalization. Our comprehensive evaluation demonstrates that QupKake outperforms all other state-of-the-art models, yielding low prediction errors on five external test sets, with RMSEs between 0.5 and 0.8 p*K*_a_ units.

Further analysis of QupKake’s feature importance revealed the crucial role of QM features, such as the coordination number and Wiberg bond order, in both reaction site enumeration and micro-p*K*_a_ prediction models. Additionally, topological features, including atom and bond types, were also found to be essential for the model’s performance.

While QupKake exhibits remarkable accuracy and generalization, we also investigated its speed and identified the tautomer search and GFN2-xTB calculations as the most time-consuming steps in the workflow. To address this challenge, we have outlined several promising research directions, including developing a faster replacement for GFN2-xTB calculations and utilizing ML models to predict certain atomic and bond features.

We believe that QupKake represents a significant contribution to the field of computational chemistry, offering a powerful tool for predicting micro-p*K*_a_ values of organic molecules. Its potential applications span a wide range of fields, including drug discovery and materials science. Moreover, the use of transfer learning, using abundant computed predictions to train an initial model, followed by experimental refinement, offers a clear mechanism to improve model accuracy in chemistry, when accurate experimental data may be scarce.

The version of QupKake used in this study and a guide to reproducing the results are available on GitHub: https://github.com/hutchisonlab/QupKake.

## Data Availability

The authors declare that the data supporting the findings of this study are available within the paper and its Supporting Information.

## Supporting Information Available

Distributions of the training and test sets, comparison of the protonation and deprotonation site models, model feature importance, model hyperparameters, chemical depictions of best and worst model p*K*_a_ predictions, and computational time required (PDF)
