# Supplementary material for: QupKake: Integrating Machine Learning and Quantum Chemistry for Micro-pKa Predictions
Source: J Chem Theory Comput. 2024 Jun 4;20(15):6946–56. doi: 10.1021/acs.jctc.4c00328 (PMC11325546; doi:10.1021/acs.jctc.4c00328)
Supplement: Supplementary file 1 — ct4c00328_si_001.pdf [file ct4c00328_si_001.pdf]

Supplementary Information for: QupKake Integrating Machine Learning and Quantum Chemistry for micro-pKa Predictions

Omri D. Abarbanel† and Geoffrey R. Hutchison*,†,‡

†*Department of Chemistry, University of Pittsburgh, 219 Parkman Avenue, Pittsburgh, Pennsylvania 15260, United States*

‡*Department of Chemical and Petroleum Engineering, University of Pittsburgh, 3700 O'Hara Street, Pittsburgh, Pennsylvania 15261, United States*

E-mail: geoffh@pitt.edu

List of Figures

ChEMBL Set Molecular Descriptions S4

ChEMBL Set pK_a Distribution S5

Experimental Set Molecular Descriptions S6

Experimental Set pK_a Distribution S7

Test Sets Molecular Descriptions S8

Test Sets pK_a Distribution S9

Reaction Sites Atoms S10

Protonation Sites Comparison S11

Deprotonation Sites Comparison S12

Reaction Enumeration Sites Model Results S15

Protonation Sites Enumeration Model Feature Importance S16

Deprotonation Sites Enumeration Model Feature Importance S17

Micro-pK_a Prediction Model Architecture S18

Test Sets Tanimoto Similarity vs. pK_a Error S20

Prediction results of low similarity test datasets S21

Prediction results on test datasets with Marvin indices S22

Prediction results on test datasets without transfer learning S23

Prediction results on test datasets trained on experimental training set S24

Best Micro-pK_a Predictions S32

Worst Micro-pK_a Predictions S33

Micro-pK_a Prediction Model Feature Importance S34

Compute Time by Step S35

Compute Time Speedup S36

List of Tables

Graph Neural Network Features . . . S13

Reaction Sites Models Hyperparameters . . . S14

Micro-pK_a Prediction Model Hyperparameters . . . S19

Set-I Nitrogen-containing aromatic heterocycles . . . S25

Set-I Aliphatic alcohols . . . S26

Set-I Aliphatic thiols . . . S26

Set-I Primary Amines . . . S27

Set-I Secondary Amines 1 . . . S27

Set-I Secondary Amines 2 . . . S27

Set-I Carboxylic Acids . . . S28

Set-I Thiophenols . . . S28

Set-I Phenols . . . S29

Set-I Anilines . . . S30

Set-I Benzoic Acids . . . S31

Set-I Carbon Acids . . . S31

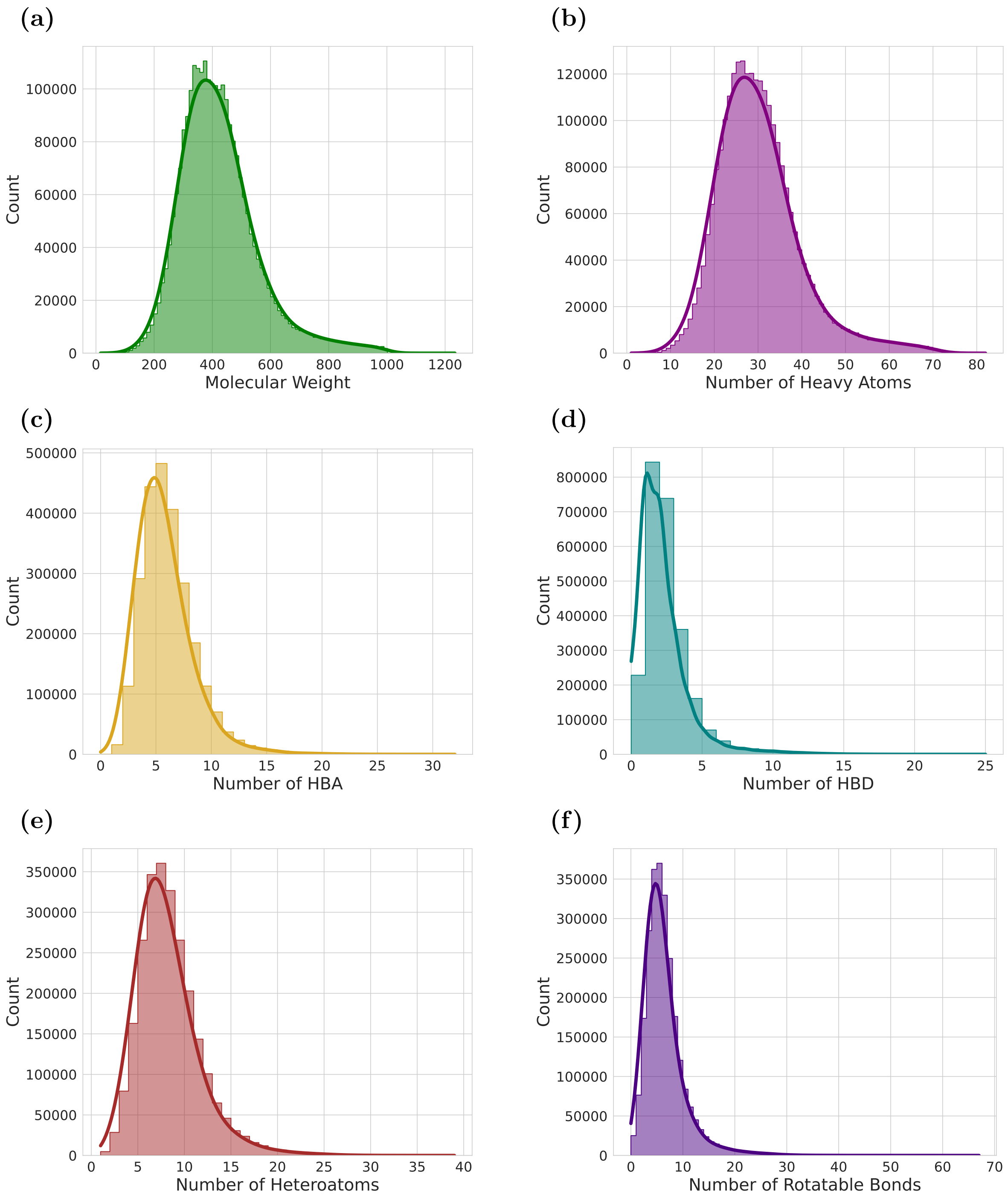

Figure S1: Molecular descriptors for the ChEMBL dataset.

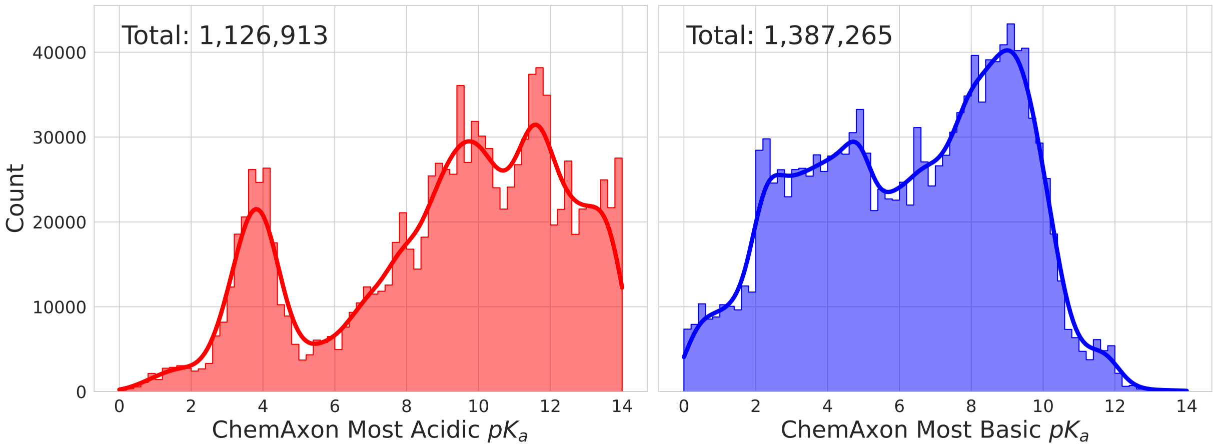

Figure S2: ChemAxon acidic and basic pK_a distribution in the ChEMBL dataset.

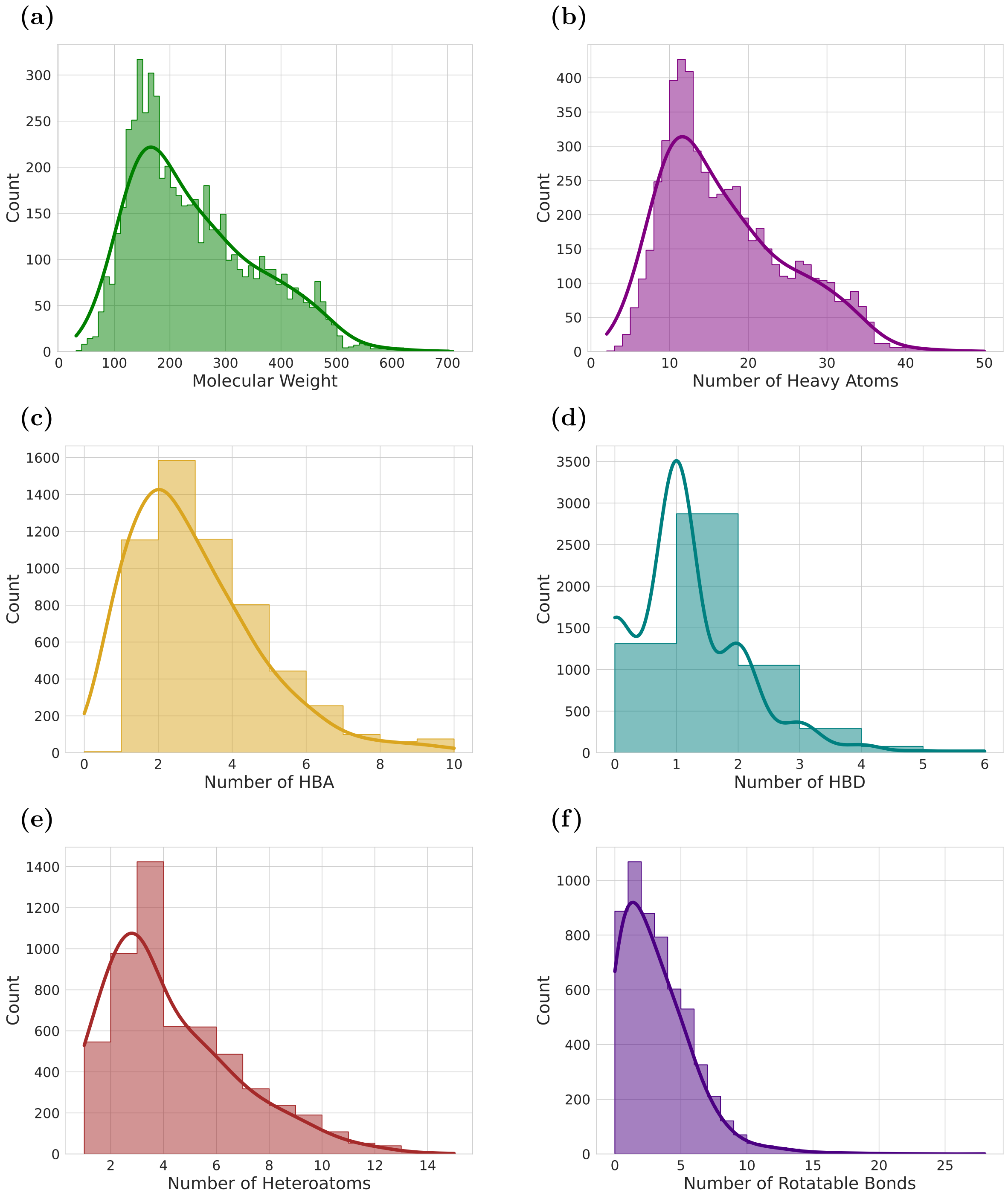

Figure S3: Molecular descriptors for the experimental dataset.

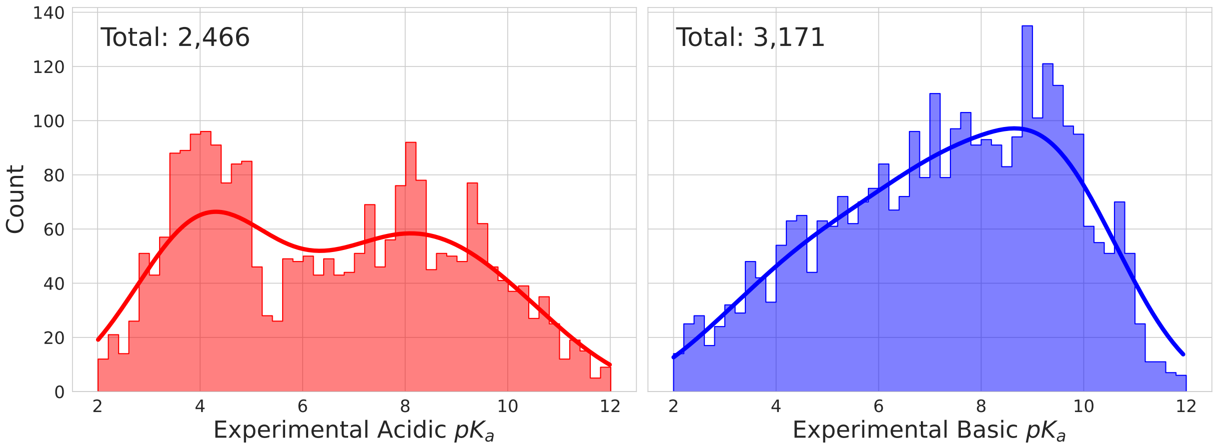

Figure S4: Acidic and basic pK_a distribution in the experimental dataset.

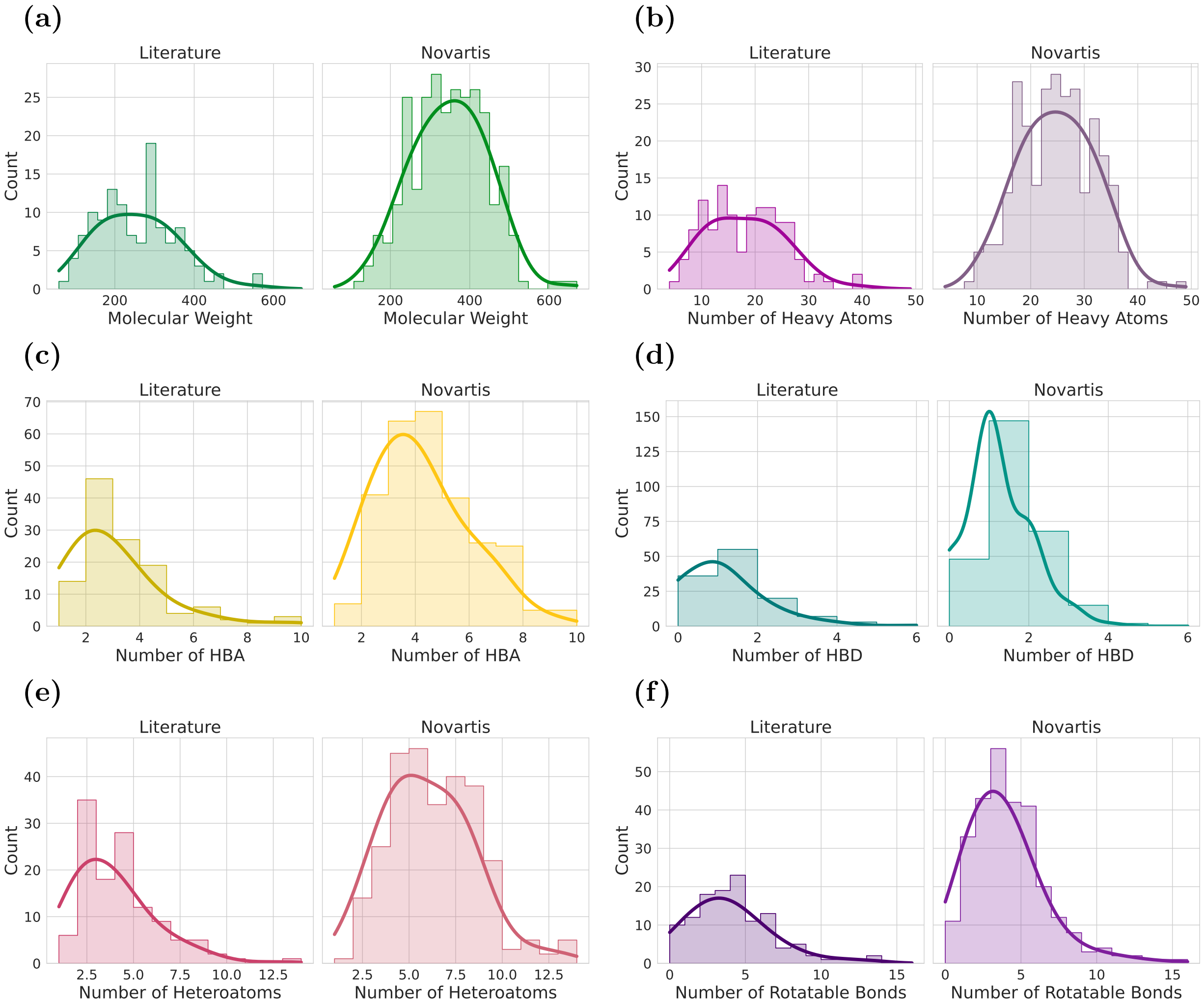

Figure S5: Molecular descriptors for the Literature and Novartis test datasets.

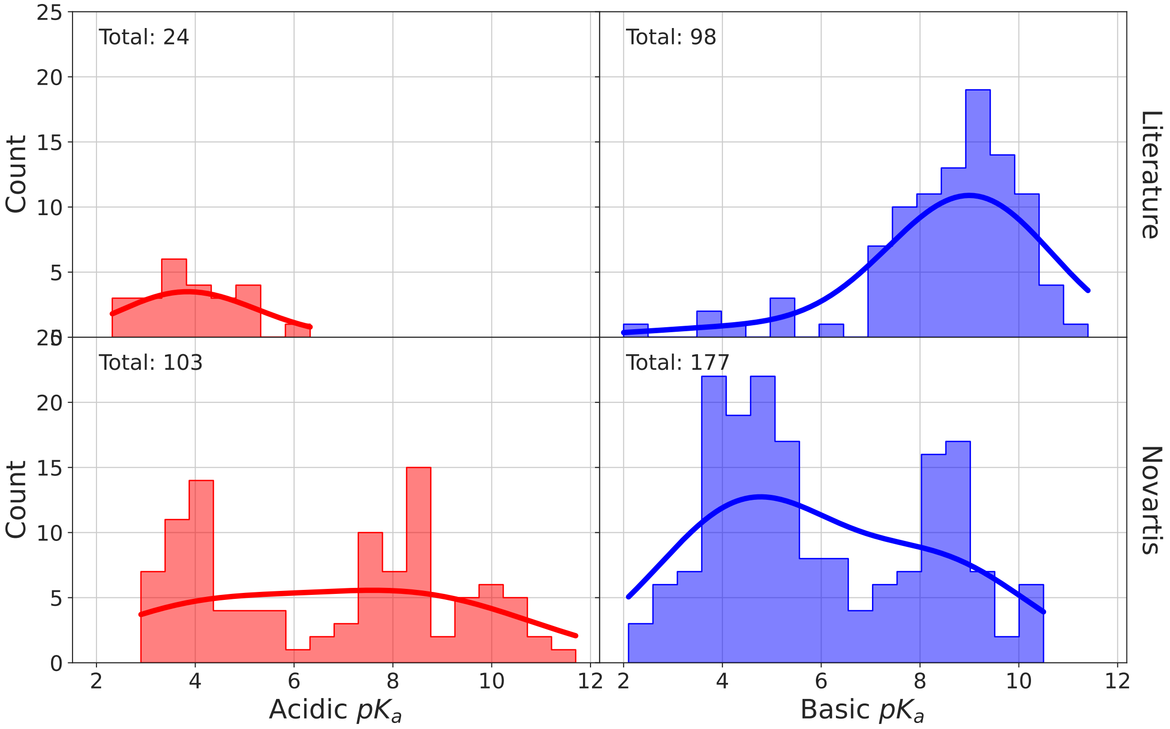

Figure S6: Experimental acidic and basic pK_a distribution in the Litarature (top row) and Novartis (bottom row) test datasets.

(a)

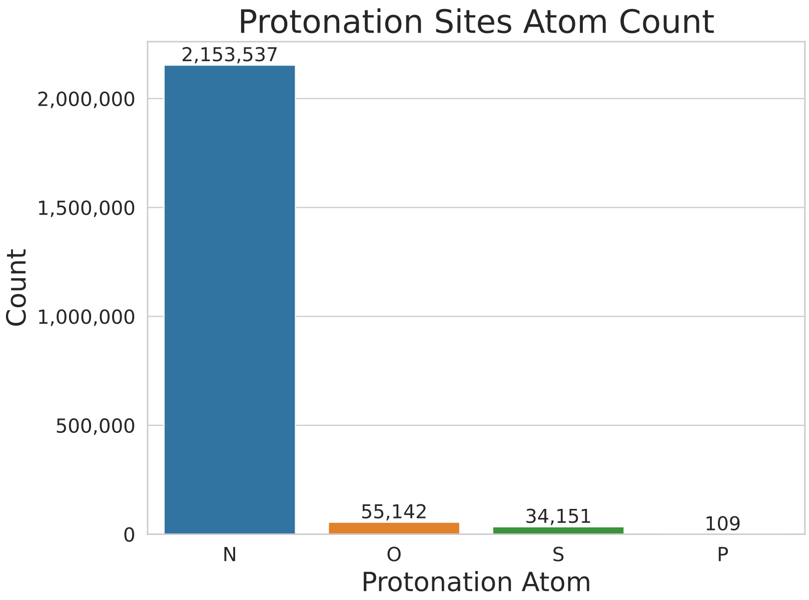

(b)

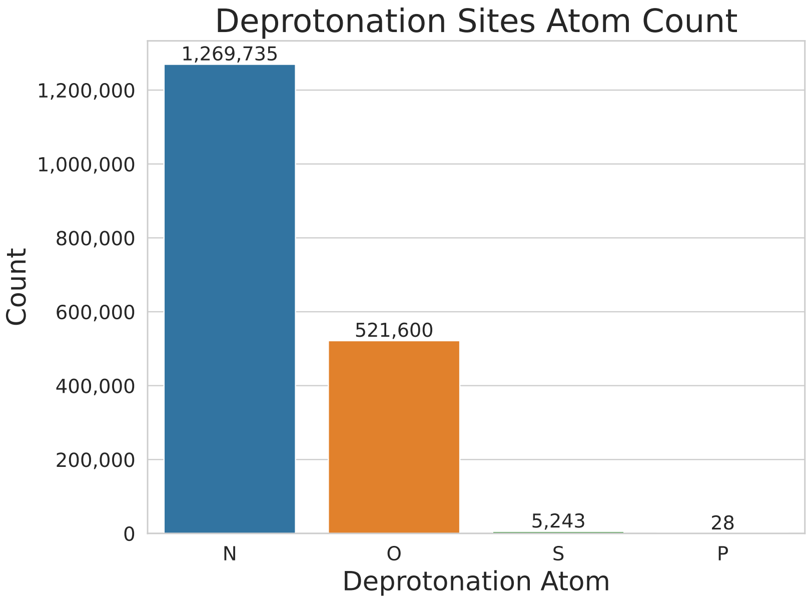

Figure S7: The number of each element that was (a) protonated or (b) deprotonated in the CREST datasets.

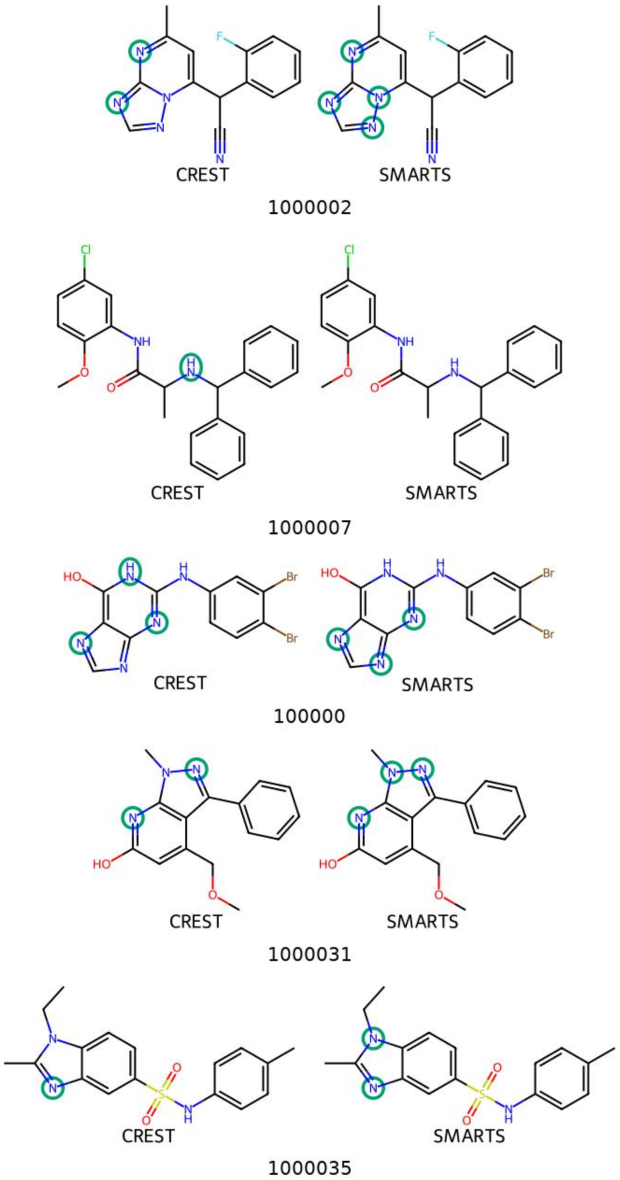

Figure S8: Examples of protonation site discrepancies between CREST and SMARTS patterns. Each row shows the same molecules with the highlighted atoms on the left show the CREST protonation sites while the right shows the SMARTS protonation sites. The Number under each pair of molecules is the ChEMBL ID.

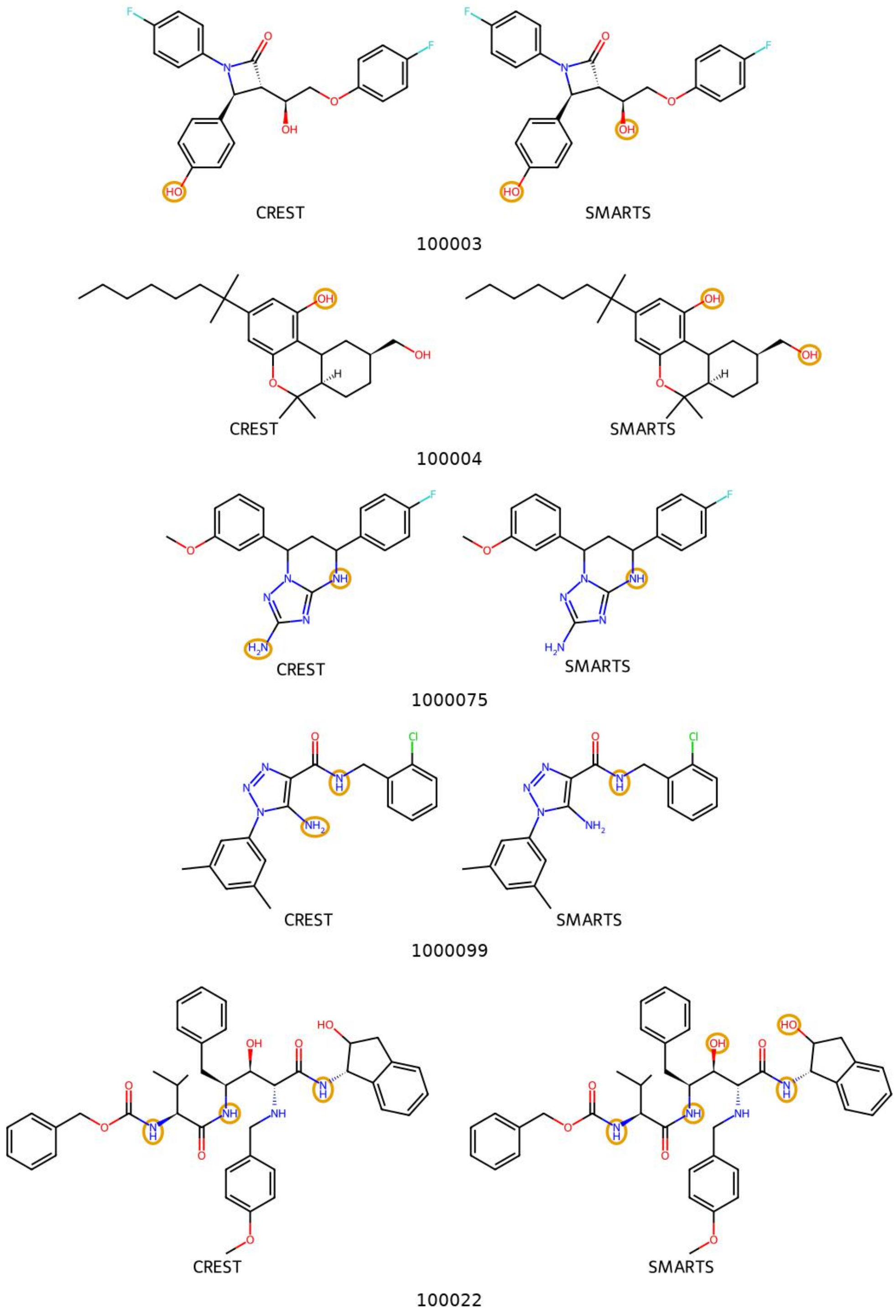

Figure S9: Examples of deprotonation site discrepancies between CREST and SMARTS patterns. Each row shows the same molecules with the highlighted atoms on the left show the CREST deprotonation sites while the right shows the SMARTS deprotonation sites. The Number under each pair of molecules is the ChEMBL ID.

Table S1: The molecular graph features used in the model.

Source	Feature	Value	Length
	**Atom Features**		
RDKit	Atom Type	One-hot encoding	17
	Heavy Atom Neighbors	One-hot encoding	6
	Formal Charge	One-hot encoding	8
	Hybridization	One-hot encoding	7
	Is In Ring	Binary	1
	Is Aromatic	Binary	1
	Atomic Mass	Float	1
	Van-Der Waals Radius	Float	1
	Covalent Radius	Float	1
	Chirality	One-hot encoding	4
	Number of Hydrogens	One-hot encoding	6
	Is H ydrogen-Bond Donor	Binary	1
	Is Hydrogen-Bond Acceptor	Binary	1
GFN2	Partial Charge	Float	1
	Coordination Number	Float	1
	Polarizability	Float	1
	Fukui Indices	Float	3
	**Total**		**61**
	**Bond Features**		
RDKit	Bond Type	One-hot encoding	4
	Is Conjugated	Binary	1
	Is In Ring	Binary	1
	Stereochemistry	One-hot encoding	4
GFN2	Wiberg Bond Order	Float	1
	**Total**		**11**
	**Molecule Features**		
RDKit	Radius of Gyration	Float	1
	Spherocity	Float	1
	Aspherocity	Float	1
	Eccentricity	Float	1
	Fraction sp^3 Carbons	Float	1
GFN2	$\Delta E_{ionization}$	Float	1
	Charge	Float	1
	**Total**		**7**

Table S2: Tuned hyperparameters for the reaction sites models, as found by Optuna. Both protonation and deprotonation models use the same hyperparameters.

Hyperparameter	Possible Values	Selected Value
GNN Architecture	GCNNet, GATNet, TransformetNet	TransformetNet
Num. of Attention Heads †	[1, 2, 3, 4]	2
Hidden Layer Size	64-512	94
Number of GNN Layers	[1, 2, 3, 4]	2

† Num. of Attention Heads parameter is only used for the GATNet and TransformerNet architectures.

(a)

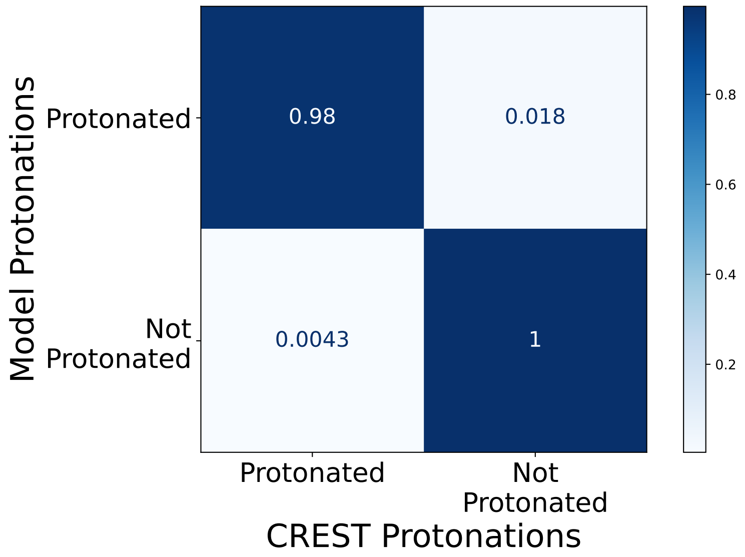

(b)

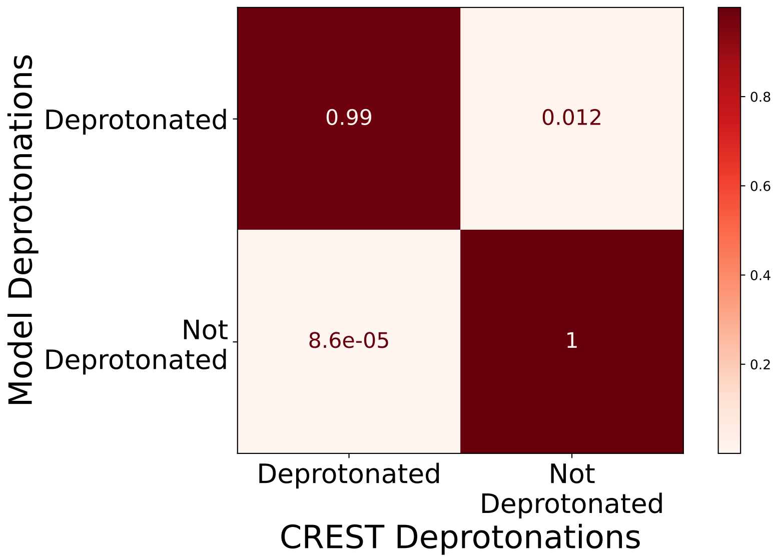

Figure S10: Confusion matrices for the (a) protonation and (b) deprotonation reaction sites enumeration models.

(a)

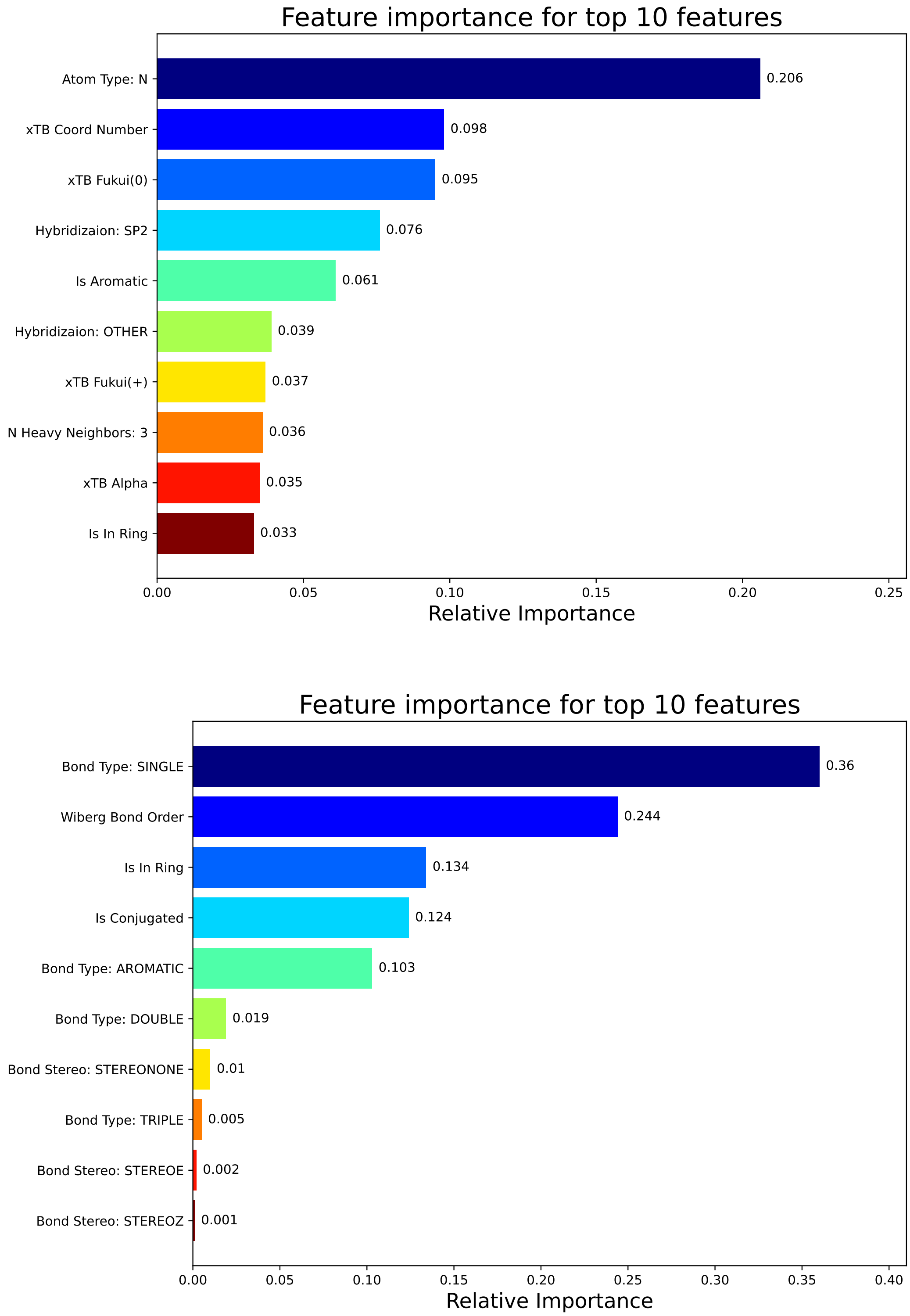

Figure S11: The normalized, absolute relative importance of the (a) atomic features and (b) bond features for the protonation sites enumeration model.

(a)

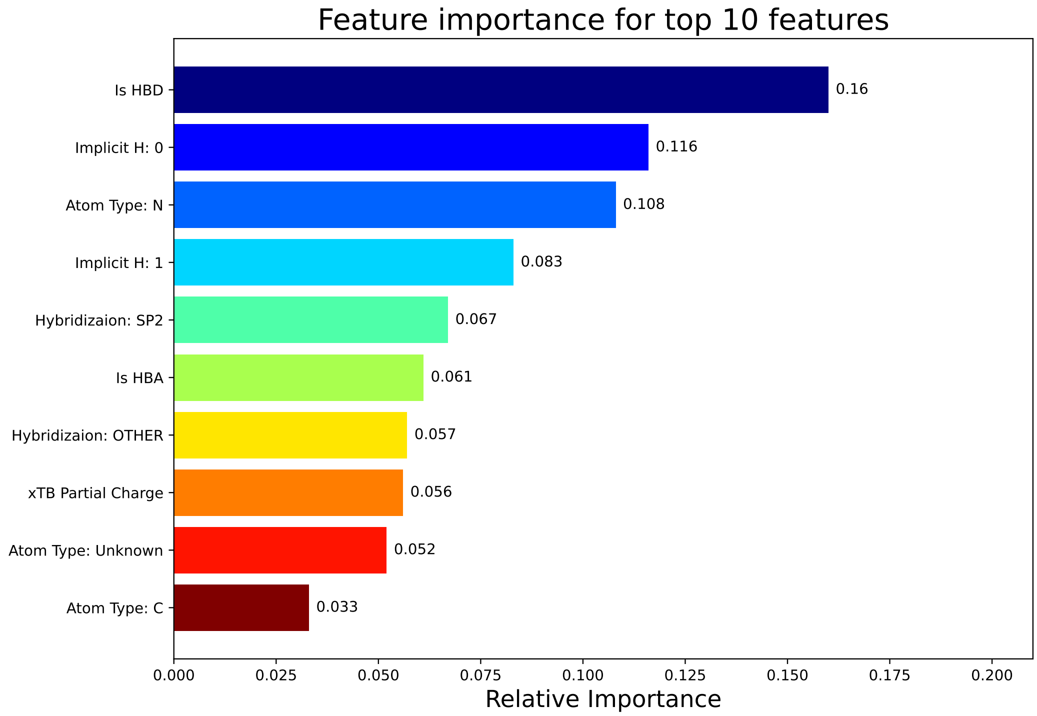

(b)

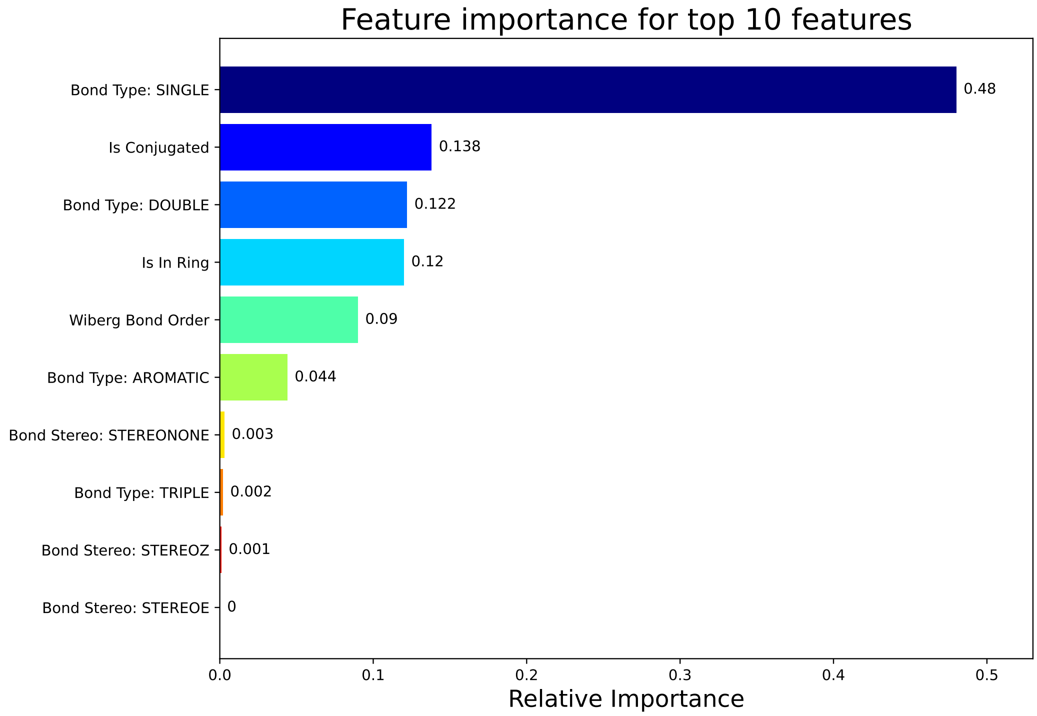

Figure S12: The normalized, absolute relative importance of the (a) atomic features and (b) bond features for the deprotonation sites enumeration model.

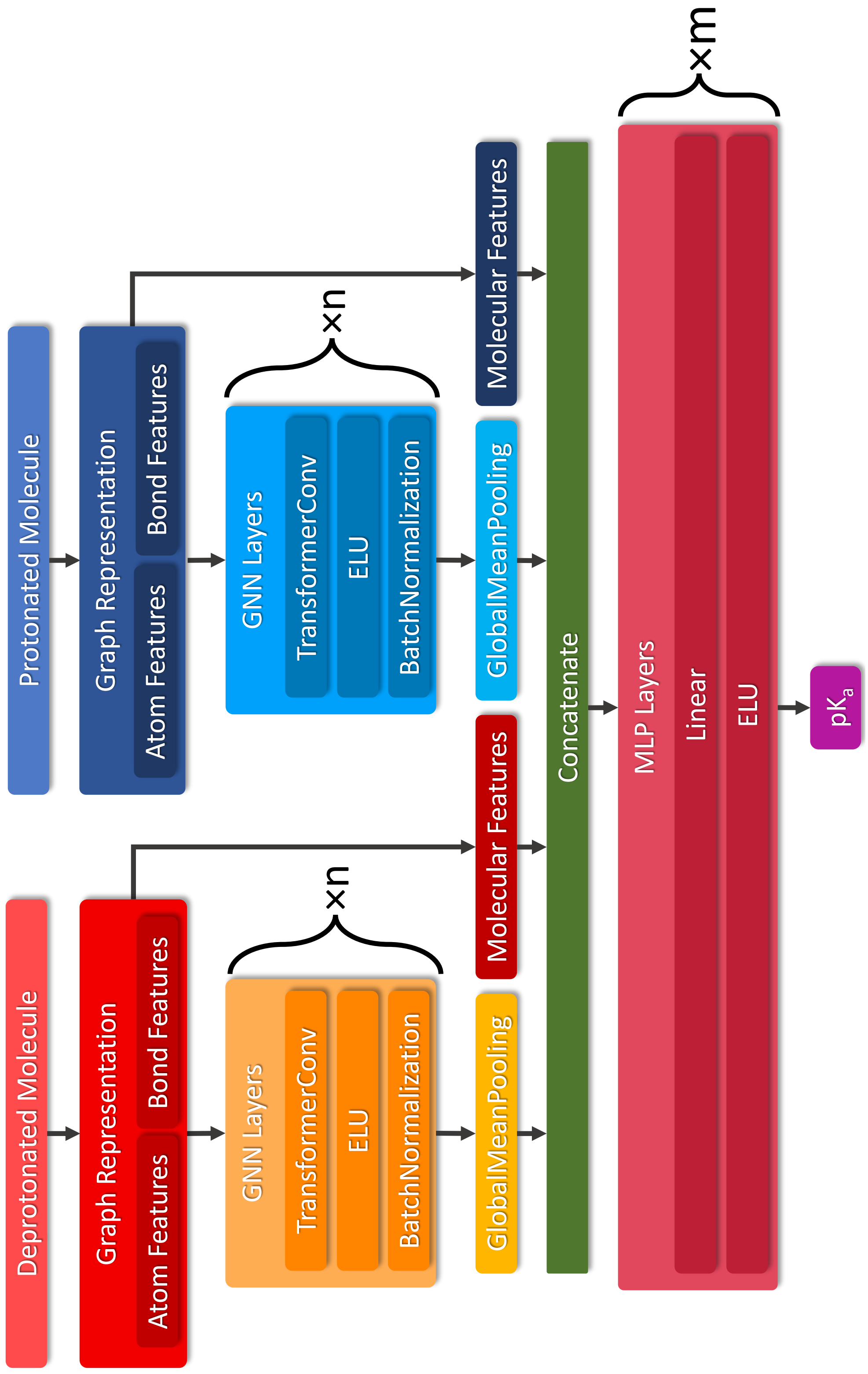

Figure S13: Micro-pK_a prediction model architecture. The parameters n and m correspond to the *Number of GNN Layers* and *Number of MLP Layers*, respectively, in Table S3.

Table S3: Tuned hyperparameters for the micro-pK_a prediction model, as found by Optuna.

Hyperparameter	Possible Values	Selected Value
GNN Architecture	GCNNet, GATNet, TransformetNet	TransformetNet
Number of Attention Heads †	[1, 2, 3, 4]	3
Hidden Layer Size	64-512	51
Number of GNN Layers	[1, 2, 3, 4]	3
Number of MLP Layers	[1, 2, 3, 4]	1

† Number of Attention Heads parameter is only used for the GATNet and TransformerNet architectures.

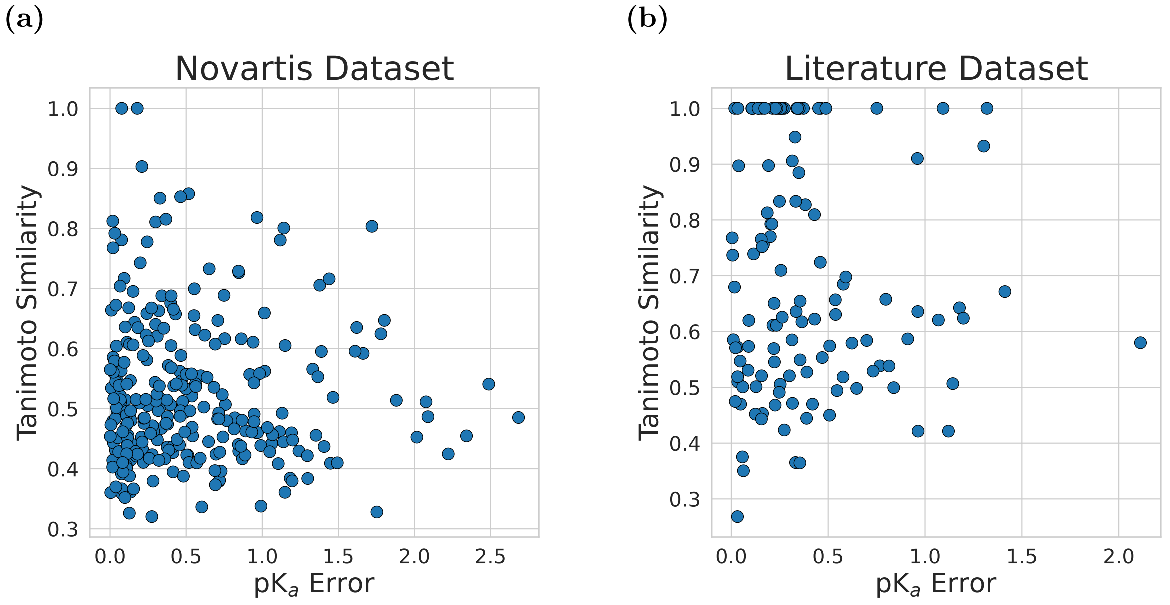

Figure S14: The highest Tanimoto similarity score of the (a) Novartis test set and the (b) Experimental test set, compared to the transfer training set, versus the pK$_a$ error, i.e. the absolut difference between the experimantal and predicts pK$_a$ values. As can be seen, there is no obvious correlation, indicating that the existence of a similar molecule in the training set has a negligible effect on the models' pK$_a$ prediction.

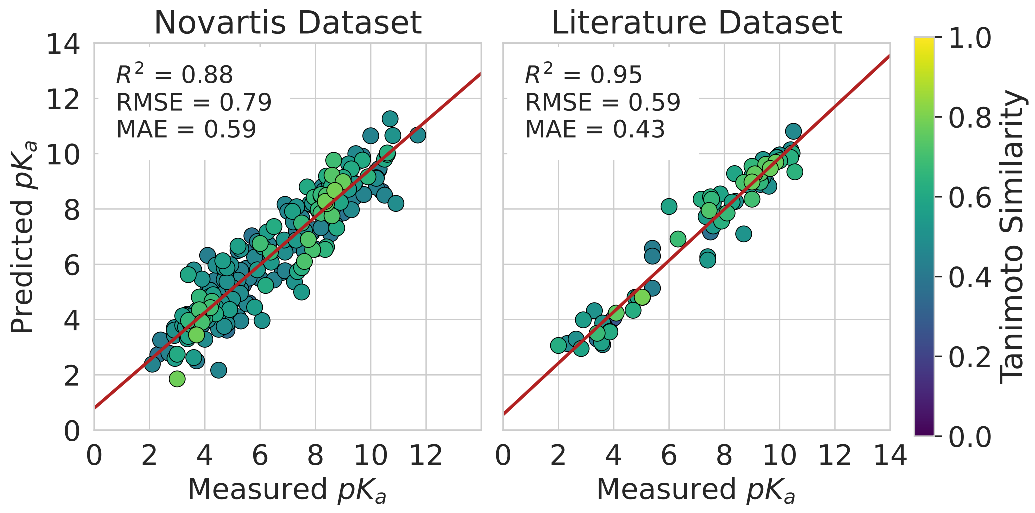

Figure S15: Micro-pK_a predictions versus the measured micro-pK_a values of the Novartis and Literature datasets, filtered to include only molecules with low (< 0.8) Taminoto similarity scores. Data points are colored according to the highest Tanimoto similarity score of the molecule in the test set versus the molecules in the experimental training set. The best-fit linear regression line is shown in red.

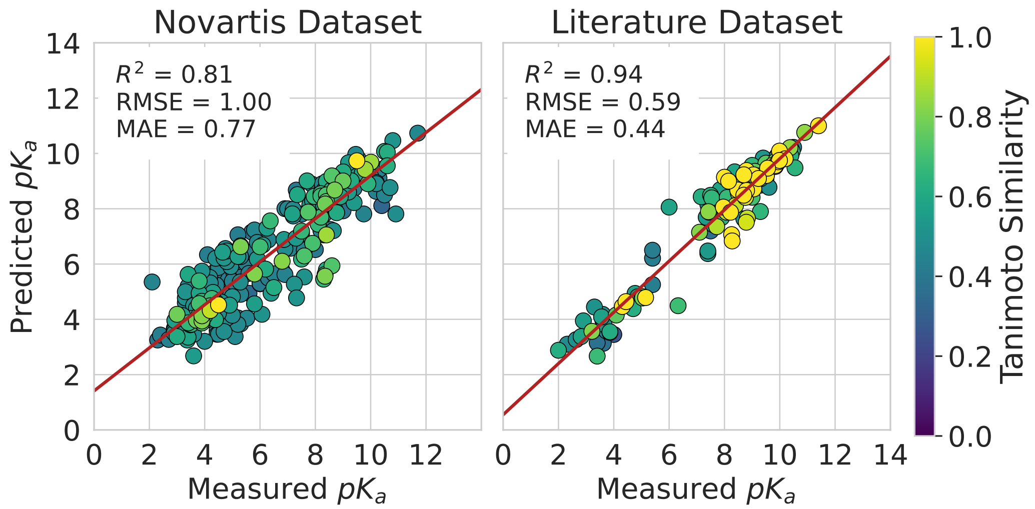

Figure S16: Micro-pK_a predictions versus the measured micro-pK_a values of the Novartis and Literature datasets, using the ChemAxon Marvin predicted reaction centers. Data points are colored according to the highest Tanimoto similarity score of the molecule in the test set versus the molecules in the experimental training set. The best-fit linear regression line is shown in red.

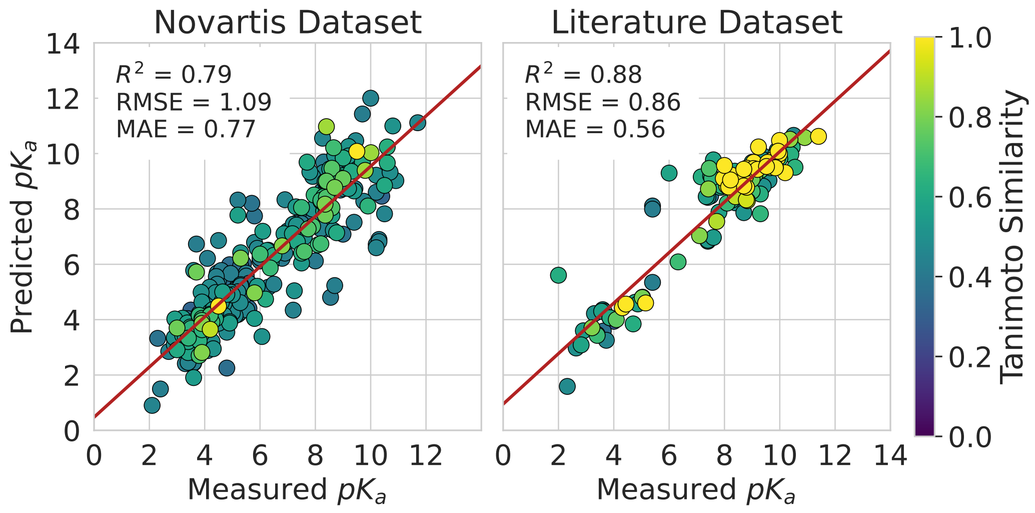

Figure S17: Micro-pK_a predictions versus the measured micro-pK_a values of the Novartis and Literature datasets, using a model trained only on the ChEMBL dataset without transfer learning. Data points are colored according to the highest Tanimoto similarity score of the molecule in the test set versus the molecules in the experimental training set. The best-fit linear regression line is shown in red.

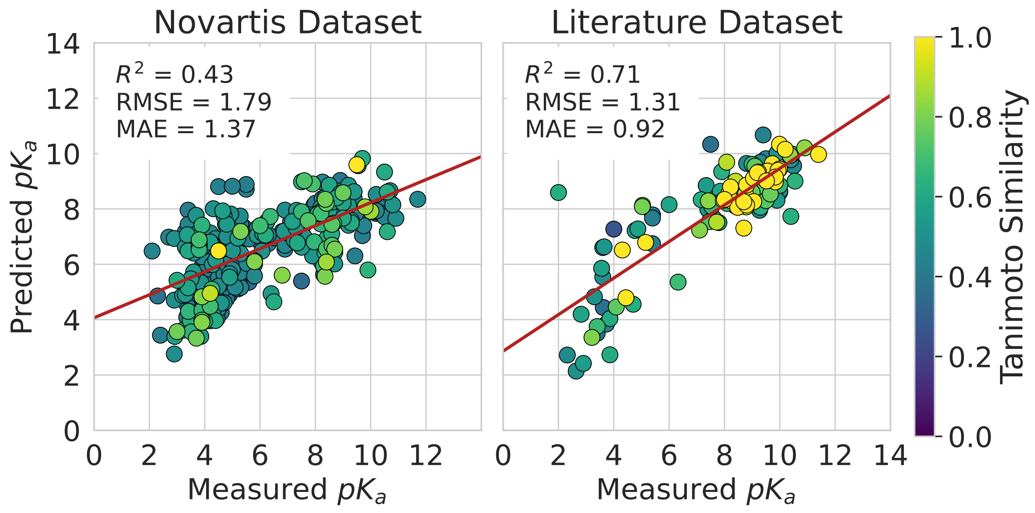

Figure S18: Micro-pK_a predictions versus the measured micro-pK_a values of the Novartis and Literature datasets, using a model trained only on the experimental dataset. Data points are colored according to the highest Tanimoto similarity score of the molecule in the test set versus the molecules in the experimental training set. The best-fit linear regression line is shown in red.

Table S4: Predictions on Nitrogen-containing aromatic heterocycles from the Thapa & Raghavachari **Set-I** dataset.[1]

			SMD only		SMD + 1 water		QupKake	
S.N.	SMILES	pK_a^{Exp}	pK_a	ΔpK_a	pK_a	ΔpK_a	pK_a	ΔpK_a
1	c1ccncn1	1.10	-0.596	-1.696	0.439	-0.661	2.161	1.061
2	c1cccnn1	2.10	0.909	-1.191	0.927	-1.173	3.185	1.085
3	c1c(cccn1)Cl	2.84	0.570	-2.270	1.828	-1.012	3.016	0.176
4	c1(ccccn1)OC	3.28	2.060	-1.220	2.627	-0.653	3.147	-0.133
5	c1cc2c(cc1)cnnc2	3.39	2.506	-0.884	2.426	-0.964	3.460	0.070
6	c1cc2c(cc1)cccn2	4.85	3.626	-1.224	4.126	-0.724	4.604	-0.246
7	c1c(cccn1)O	4.86	3.140	-1.720	3.926	-0.934	4.454	-0.406
8	c1ccc(cn1)OC	4.88	3.412	-1.468	4.192	-0.688	4.777	-0.103
9	c1cc2c(cc1)cc1c(c2)nccc1	5.05	3.525	-1.525	4.046	-1.004	4.268	-0.782
10	c1ccccn1	5.17	3.838	-1.332	4.323	-0.847	4.881	-0.289
11	c1cc2c(cc1)cc1c(cccc1)n2	5.60	4.425	-1.175	4.483	-1.117	4.801	-0.799
12	c1c(cccn1)CC	5.70	4.382	-1.318	5.106	-0.594	5.282	-0.418
13	c1c(cccn1)C(C)(C)C	5.82	4.499	-1.321	5.038	-0.782	5.433	-0.387
15	c1cccc(n1)CC	5.97	5.081	-0.889	5.247	-0.723	5.643	-0.327
16	c1cc(ccn1)C(C)(C)C	5.99	4.729	-1.261	5.339	-0.651	5.646	-0.344
17	c12c(cccc1)nc[nH]2	6.00	3.650	-2.350	4.942	-1.058	5.038	-0.962
18	c1cc(ccn1)CC	6.02	4.745	-1.275	5.328	-0.692	5.848	-0.172
19	c1[nH]cc(n1)CO	6.45	4.921	-1.529	5.613	-0.837	5.979	-0.471
20	c1cc(ccn1)OC	6.62	5.326	-1.294	5.558	-1.062	6.243	-0.377
21	c1(cnc[nH]1)C[C@@H](C(=O)[O])NC(=O)C	7.05	6.293	-0.757	7.053	0.003	6.100	-0.950
22	c1(ncc[nH]1)C	7.75	7.148	-0.602	8.010	0.260	7.482	-0.268
	MAE			1.348		0.783		0.468
	MSE			1.41		0.829		0.568
	MaxAbsError			2.350		1.173		1.085

Table S5: Predictions on Aliphatic alcohols from the Thapa & Raghavachari **Set-I** dataset.[1]

			SMD only		SMD + 1 water		SMD + 2 waters		SMD + 3 waters		QupKake	
S.N.	SMILES	pK_a^{Exp}	pK_a	ΔpK_a	pK_a	ΔpK_a	pK_a	ΔpK_a	pK_a	ΔpK_a	pK_a	ΔpK_a
1	C(C(O)(C)C)(F)(F)F	11.60	20.692	9.092	18.025	6.425	14.767	3.167	12.591	0.991	2.372	-9.228
2	C(CO)(Cl)(Cl)Cl	12.02	18.854	6.834	15.877	3.857	13.017	0.997	11.466	-0.554	0.592	-11.428
3	C(CO)(F)(F)F	12.43	19.877	7.447	16.966	4.536	14.217	1.787	12.047	-0.383	2.584	-9.846
4	C#CCO	13.55	22.443	8.893	18.858	5.308	16.579	3.029	13.663	0.113	2.663	-10.887
5	C(O)COC	14.80	24.311	9.511	20.673	5.873	17.643	2.843	15.187	0.387	5.995	-8.805
6	C(O)C[C@H](O)C	14.90	25.718	10.818	21.410	6.510	19.038	4.138	15.396	0.496	7.968	-6.932
7	C(O)CCO	15.10	25.600	10.500	21.491	6.391	18.603	3.503	15.528	0.428	5.012	-10.088
8	c1ccc(cc1)CO	15.40	24.674	9.274	20.769	5.369	18.112	2.712	14.968	-0.432	2.425	-12.975
9	C(O)/C=C/C	15.52	25.482	9.962	21.257	5.737	18.422	2.902	15.817	0.297	3.424	-12.096
10	CO	15.54	26.118	10.578	21.838	6.298	18.880	3.340	16.474	0.934	10.409	-5.131
11	C(O)C	15.90	25.961	10.061	21.797	5.897	18.407	2.507	16.095	0.195	9.918	-5.982
12	C(O)CC	16.10	26.072	9.972	21.758	5.658	18.971	2.871	15.961	-0.139	4.261	-11.839
13	C1CCC(CC1)O	16.84	26.543	9.703	22.169	5.329	19.438	2.598	17.280	0.440	3.964	-12.876
14	C(O)(C)(C)C	17.00	26.515	9.515	22.251	5.251	19.432	2.432	17.028	0.028	8.990	-8.010
15	C(O)(C)C	17.10	26.167	9.067	21.903	4.803	19.649	2.549	16.996	-0.104	8.596	-8.504
	MAE			9.415		5.549		2.758		0.395		9.642
	MSE			9.474		5.597		2.846		0.479		0.991
	MaxAbsError			10.818		6.510		4.138		0.991		12.975

Table S6: Predictions on Aliphatic thiols from the Thapa & Raghavachari **Set-I** dataset.[1]

			SMD only		SMD + 1 water		SMD + 2 waters		SMD + 3 waters		QupKake	
S.N.	SMILES	pK_a^{Exp}	pK_a	ΔpK_a	pK_a	ΔpK_a	pK_a	ΔpK_a	pK_a	ΔpK_a	pK_a	ΔpK_a
1	C=CCS	7.86	18.450	10.590	14.685	6.825	12.525	4.665	8.698	0.838	4.004	-3.856
2	SCC(=O)OCC	7.95	14.675	6.725	12.120	4.170	9.128	1.178	7.377	-0.573	3.875	-4.075
3	SC[C@@H](CO)S	8.62	17.444	6.874	14.306	3.736	12.747	2.177	7.720	-0.900	4.788	-3.832
4	C(OCC)CS	9.38	17.947	8.567	14.398	5.018	11.887	2.507	8.553	-0.827	3.624	-5.756
5	OCCS	9.72	18.114	8.394	14.788	5.068	12.147	2.427	9.515	-0.205	4.354	-5.366
6	SC(C)(C)CO	9.85	17.777	7.927	13.492	3.642	11.539	1.689	8.971	-0.879	3.793	-6.057
7	C(=C)CS	9.96	17.945	7.985	14.643	4.683	11.992	2.032	9.263	-0.697	3.865	-6.095
8	C(C(=O)[O])CS	10.27	17.468	7.198	13.672	3.402	13.946	3.676	11.332	1.062	3.404	-6.866
9	SC	10.33	19.557	9.227	15.986	5.656	13.348	3.018	10.147	-0.183	7.192	-3.138
10	CCS	10.61	19.589	8.979	16.046	5.436	13.238	2.628	10.545	-0.065	6.536	-4.074
11	C(CC)CS	10.67	19.752	9.082	15.772	5.102	13.533	2.863	10.508	-0.162	4.402	-6.268
12	SC(C)C	10.86	19.516	8.656	16.403	5.543	13.397	2.537	10.695	-0.165	5.795	-5.065
13	SC(C)(C)C	11.05	19.882	8.832	15.999	4.949	13.729	2.679	11.005	-0.045	6.480	-4.570
14	SC(C)(C)CC	11.22	19.994	8.774	16.508	5.288	14.069	2.849	10.770	-0.450	5.037	-6.183
	MAE			8.554		5.033		2.777		0.504		5.086
	MSE			8.601		5.104		2.913		0.612		5.207
	MaxAbsError			10.59		6.825		4.665		1.062		6.866

Table S7: Predictions on primary amines from the Thapa & Raghavachari **Set-I** dataset.[1]

			SMD only		SMD + 1 water		SMD + 2 waters		SMD + 3 waters		QupKake	
S.N.	SMILES	pK_a^{Exp}	pK_a	ΔpK_a	pK_a	ΔpK_a	pK_a	ΔpK_a	pK_a	ΔpK_a	pK_a	ΔpK_a
1	NCC#N	5.30	3.245	-2.055	3.510	-1.790	4.896	-0.404	5.883	0.583	5.254	-0.046
2	c1cc(ccc1)CN	9.34	9.029	-0.311	8.914	-0.426	10.114	0.774	11.268	1.928	8.747	-0.593
3	c1cc(ccc1)CCN	9.68	9.138	-0.542	8.728	-0.952	9.656	-0.024	10.694	1.014	9.399	-0.281
4	C(N)(C)C	9.80	10.595	0.795	9.814	0.014	10.431	0.631	11.290	1.490	9.880	0.080
5	NCCCC	10.59	10.334	-0.256	9.463	-1.127	10.269	-0.321	11.268	0.678	10.014	-0.576
6	C(CN)C	10.60	10.481	-0.119	9.725	-0.875	10.466	-0.134	11.547	0.947	9.998	-0.602
7	CN	10.63	10.193	-0.437	9.585	-1.045	10.457	-0.173	10.782	0.152	11.484	0.854
8	C(N)(C)(C)C	10.68	10.833	0.153	9.911	-0.769	10.895	0.215	12.004	1.324	10.336	-0.344
9	C(N)C	10.70	10.258	-0.442	9.668	-1.032	10.622	-0.078	11.098	0.398	9.923	-0.777
10	[C@@H]1(CC[C@@H](CC1)N)C(C)(C)C	11.23	10.879	-0.351	10.165	-1.065	10.643	-0.587	12.900	1.670	10.693	-0.537
	MAE			0.546		0.910		0.334		1.018		0.469
	MSE			0.765		1.010		0.413		1.157		0.536
	MaxAbsError			2.055		1.79		0.774		1.928		0.854

Table S8: Predictions on secondary amines I from the Thapa & Raghavachari **Set-I** dataset.[1]

			SMD only		SMD + 1 water		SMD + 2 waters		QupKake	
S.N.	SMILES	pK_a^{Exp}	pK_a	ΔpK_a	pK_a	ΔpK_a	pK_a	ΔpK_a	pK_a	ΔpK_a
1	N(C[C@@H](c1ccc(c(c1)O)O)O)C	8.55	8.446	-0.104	8.925	0.375	10.520	1.970	8.912	0.362
2	C(NC)C	10.54	10.749	0.209	10.658	0.118	12.211	1.671	10.038	-0.502
3	CNC	10.78	10.288	-0.492	10.494	-0.286	11.870	1.090	10.206	-0.574
4	CCCNCCC	11.00	11.106	0.106	10.876	-0.124	12.148	1.148	10.560	-0.440
5	C(NCC)C	11.02	11.161	0.141	10.748	-0.272	12.189	1.169	10.000	-1.020
6	C1CCNCC1	11.22	10.864	-0.356	10.791	-0.429	11.696	0.476	10.489	-0.731
7	C(CNC1CCCCC1)C	11.23	11.286	0.056	11.362	0.132	11.903	0.673	11.897	0.667
8	C1CCCN1	11.27	10.447	-0.823	10.605	-0.665	11.766	0.496	10.943	-0.327
	MAE			0.286		0.300		1.087		0.578
	MSE			0.377		0.348		1.197		0.616
	MaxAbsError			0.823		0.665		1.970		1.020

Table S9: Predictions on secondary amines II from the Thapa & Raghavachari **Set-I** dataset.[1]

			SMD only		SMD + 1 water		QupKake	
S.N.	SMILES	pK_a^{Exp}	pK_a	ΔpK_a	pK_a	ΔpK_a	pK_a	ΔpK_a
S.N.	SMILES	pK_a^{Exp}	pK_a	ΔpK_a	pK_a	ΔpK_a	pK_a	ΔpK_a
1	C1CCN(CC1)CC=C	9.69	9.693	0.003	10.160	0.470	9.112	-0.578
2	C1CCN(CC1)CC=C	9.69	9.987	0.297	10.172	0.482	9.071	-0.619
3	CN(C)C	9.80	9.641	-0.159	10.802	1.002	9.126	-0.674
4	C(N(C)C)C	10.16	10.218	0.058	10.797	0.637	9.606	-0.554
5	CCN(CC)CC	10.75	11.490	0.740	11.173	0.423	10.097	-0.653
	MAE			0.251		0.603		0.616
	MSE			0.365		0.639		0.617
	MaxAbsError			0.740		1.002		0.674

Table S10: Predictions on carboxylic acids from the Thapa & Raghavachari **Set-I** dataset.[1]

			SMD only		SMD + 1 water		SMD + 2 waters		SMD + 3 waters		QupKake	
S.N.	SMILES	pK_a^{Exp}	pK_a	ΔpK_a	pK_a	ΔpK_a	pK_a	ΔpK_a	pK_a	ΔpK_a	pK_a	ΔpK_a
1	C(=O)(O)C(F)(F)F	-0.26	-1.526	-1.266	-0.088	0.172	-0.267	-0.007	-0.668	-0.408	2.101	2.361
2	C(=O)(O)C(Cl)(Cl)Cl	0.65	-1.123	-1.772	-0.014	-0.664	-0.015	-0.665	-0.817	-1.467	1.445	0.795
3	C(=O)(O)C(F)F	1.24	0.914	-0.326	1.791	0.551	1.921	0.681	0.893	-0.347	2.129	0.889
4	C(=O)(O)C(Cl)Cl	1.30	1.126	-0.174	1.924	0.624	1.539	0.239	0.705	-0.595	0.655	-0.645
5	C(#N)CC(=O)O	2.44	2.893	0.453	3.227	0.787	3.138	0.698	2.202	-0.238	2.239	-0.201
6	C(=O)(O)CF	2.66	3.533	0.873	3.726	1.066	3.266	0.606	2.615	-0.045	1.933	-0.727
7	C(=O)(O)[C@@H](Cl)C	2.80	4.636	1.836	4.276	1.476	4.235	1.435	3.114	0.314	1.666	-1.134
8	C(=O)(O)CCl	2.81	4.434	1.624	4.625	1.815	4.395	1.585	2.968	0.158	2.036	-0.774
9	C#CCC(=O)O	2.86	4.748	1.888	4.786	1.926	4.325	1.465	3.654	0.794	2.300	-0.560
10	C(=O)(O)CBr	2.86	4.094	1.234	4.367	1.507	3.871	1.011	2.74	-0.12	2.306	-0.554
11	C(=O)(O)CC(F)(F)F	3.07	4.650	1.580	4.698	1.628	4.442	1.372	3.081	0.011	2.922	-0.148
12	C(=O)(O)CC(=O)C	3.53	5.244	1.714	5.022	1.492	4.702	1.172	3.391	-0.139	2.113	-1.417
13	COCC(=O)O	3.54	4.642	1.102	4.747	1.207	4.315	0.775	3.563	0.023	2.198	-1.342
14	C(=O)O	3.75	5.333	1.583	4.774	1.024	4.658	0.908	2.707	-1.043	0.144	-3.606
15	C(=O)(O)CO	3.83	4.835	1.005	5.149	1.319	4.395	0.565	3.567	-0.263	2.206	-1.624
16	C(=O)(O)[C@H](O)C	3.87	3.607	-0.263	3.540	-0.330	3.491	-0.379	NaN	NaN	2.143	-1.727
17	C(CC(=O)O)Cl	4.10	6.168	2.068	5.697	1.597	4.843	0.743	4.361	0.261	2.083	-2.017
18	C(=O)(O)C=C	4.26	6.356	2.096	6.120	1.860	5.490	1.230	4.879	0.619	1.168	-3.092
19	c1ccccc1CC(=O)O	4.31	6.279	1.969	5.879	1.569	5.505	1.195	4.153	-0.157	1.802	-2.508
20	C=CCC(=O)O	4.35	6.376	2.026	6.035	1.685	5.337	0.987	4.777	0.427	3.397	-0.953
21	ClCCCC(=O)O	4.52	6.969	2.449	6.490	1.970	5.764	1.244	4.654	0.134	1.928	-2.592
22	C(=O)(O)C	4.76	7.718	2.958	7.173	2.413	6.896	2.136	5.378	0.618	2.254	-2.506
23	C(=O)(O)CCC	4.82	8.096	3.276	7.204	2.384	6.906	2.086	5.557	0.737	1.974	-2.846
24	C(=O)(O)CC	4.87	8.107	3.237	7.552	2.682	7.200	2.330	5.594	0.724	2.017	-2.853
25	C1CCC(CC1)C(=O)O	4.90	7.953	3.053	7.356	2.456	6.812	1.912	5.542	0.642	2.508	-2.392
26	C(=O)(O)C(C)(C)C	5.05	8.059	3.009	7.616	2.566	7.103	2.053	5.917	0.867	2.188	-2.862
	MAE			1.724		1.491		1.134		0.446		1.659
	MSE			1.943		1.642		1.285		0.569		1.931
	MaxAbsError			3.276		2.682		2.330		1.467		3.606

Table S11: Predictions on thiophenols from the Thapa & Raghavachari **Set-I** dataset.[1]

			SMD only		SMD + 1 water		SMD + 2 waters		SMD + 3 waters		QupKake	
S.N.	SMILES	pK_a^{Exp}	pK_a	ΔpK_a	pK_a	ΔpK_a	pK_a	ΔpK_a	pK_a	ΔpK_a	pK_a	ΔpK_a
1	Sc1ccc(cc1)N(=O)=O	4.72	8.622	3.902	6.254	1.534	3.743	-0.977	2.276	-2.444	2.340	-2.380
2	Sc1cc(ccc1)N(=O)=O	5.24	9.979	4.739	7.252	2.012	5.204	-0.036	2.977	-2.263	2.477	-2.763
3	Sc1c[nH]c(=O)[nH]c1=O	5.30	10.010	4.710	7.428	2.128	5.220	-0.080	2.689	-2.611	4.437	-0.863
4	Sc1ccc(cc1)C(=O)C	5.33	10.345	5.015	7.826	2.496	5.302	-0.028	3.331	-1.999	2.315	-3.015
5	Sc1cc(ccc1)Cl	5.78	11.171	5.391	8.372	2.592	5.707	-0.073	3.632	-2.148	2.582	-3.198
6	Sc1ccc(cc1)Br	6.02	11.629	5.609	8.835	2.815	6.797	0.777	4.350	-1.670	2.697	-3.323
7	Sc1ccc(cc1)Cl	6.14	11.676	5.536	9.080	2.940	6.797	0.657	4.169	-1.971	2.645	-3.495
8	c1(cc(ccc1)OC)S	6.39	12.533	6.143	9.568	3.178	7.271	0.881	4.746	-1.644	2.839	-3.551
9	Sc1ccccc1	6.61	12.643	6.033	9.933	3.323	7.225	0.615	5.170	-1.440	2.931	-3.679
10	Sc1c(cccc1)C	6.64	13.467	6.827	10.465	3.825	8.328	1.688	5.832	-0.808	2.941	-3.699
11	Sc1cc(ccc1)C	6.66	12.976	6.316	10.115	3.455	7.603	0.943	5.278	-1.382	2.860	-3.800
12	Sc1cc(ccc1)OC	6.78	12.500	5.720	9.661	2.881	7.142	0.362	4.992	-1.788	2.837	-3.943
13	Sc1ccc(cc1)C	6.82	13.226	6.406	10.454	3.634	7.798	0.978	5.557	-1.263	3.142	-3.678
	MAE			5.565		2.832		0.623		1.802		3.184
	MSE			5.620		2.904		0.784		1.867		3.281
	MaxAbsError			6.827		3.825		1.688		2.611		3.943

Table S12: Predictions on phenols from the Thapa & Raghavachari **Set-I** dataset.[1]

			SMD only		SMD + 1 water		SMD + 2 waters		SMD + 3 waters		QupKake	
S.N.	SMILES	pK_a^{Exp}	pK_a	ΔpK_a	pK_a	ΔpK_a	pK_a	ΔpK_a	pK_a	ΔpK_a	pK_a	ΔpK_a
1	c1cc(c(cc1)O)C=O	6.79	14.197	7.407	11.580	4.790	10.508	3.718	6.894	0.104	2.029	-4.761
2	c1(ccc(cc1)O)N(=O)=O	7.14	9.699	2.559	9.068	1.928	7.625	0.485	5.877	-1.263	2.321	-4.819
3	c1ccc(c(c1)N(=O)=O)O	7.23	8.498	1.268	9.411	2.181	7.253	0.023	6.013	-1.217	2.492	-4.738
4	c1c(ccc(c1)O)C=O	7.66	11.415	3.755	10.388	2.728	8.728	1.068	7.765	0.105	2.734	-4.926
5	c1c(ccc(c1)O)C#N	7.95	11.838	3.888	10.968	3.018	9.043	1.093	7.730	-0.220	1.598	-6.352
6	c1c(cc(cc1)O)C=O	8.00	14.010	6.010	12.135	4.135	10.374	2.374	8.302	0.302	1.781	-6.219
7	c1ccc(cc1N(=O)=O)O	8.35	12.447	4.097	11.516	3.166	8.966	0.616	7.285	-1.065	2.105	-6.245
8	c1cc(ccc1O)C(=O)OC/C=C/C	8.41	12.275	3.865	11.238	2.828	9.425	1.015	7.604	-0.806	1.989	-6.421
9	c1cc(ccc1O)C(=O)OC	8.47	12.299	3.829	11.295	2.825	9.270	0.800	7.870	-0.600	1.938	-6.532
10	c1cc(ccc1O)C(=O)OCCCC	8.47	12.378	3.908	11.255	2.785	9.372	0.902	8.192	-0.278	1.280	-7.190
11	Clc1ccccc1O	8.48	12.777	4.297	11.166	2.686	9.496	1.016	7.196	-1.284	1.760	-6.720
12	c1cc(ccc1O)C(=O)OCC	8.50	12.397	3.897	11.217	2.717	8.970	0.470	8.074	-0.426	1.739	-6.761
13	c1cc(cc(c1)O)C#N	8.61	13.095	4.485	11.859	3.249	9.884	1.274	8.245	-0.365	1.394	-7.216
14	c1ccc(c(c1)F)O	8.81	13.078	4.268	11.620	2.810	9.522	0.712	7.400	-1.410	1.510	-7.300
15	c1ccc(cc1Cl)O	9.02	13.634	4.614	11.814	2.794	9.753	0.733	8.063	-0.957	1.772	-7.248
16	c1ccc(cc1F)O	9.28	13.914	4.634	12.230	2.950	10.168	0.888	8.861	-0.419	1.854	-7.426
17	c1(ccc(cc1)O)Cl	9.38	14.332	4.952	13.005	3.625	10.720	1.340	9.147	-0.233	1.719	-7.661
18	c1(ccc(cc1)O)C(=O)[O]	9.39	14.885	5.495	13.626	4.236	11.218	1.828	9.553	0.163	2.642	-6.748
19	c1ccc(cc1O)O	9.44	14.985	5.545	13.233	3.793	10.986	1.546	9.373	-0.067	2.117	-7.323
20	c1ccc(c(c1)O)O	9.48	12.734	3.254	12.835	3.355	11.386	1.906	9.725	0.245	2.155	-7.325
21	c1c(ccc(c1)c1ccccc1)O	9.51	15.087	5.577	13.175	3.665	10.921	1.411	9.372	-0.138	0.033	-9.477
22	c1cc(cc(c1)c1ccccc1)O	9.59	15.351	5.761	13.279	3.689	10.889	1.299	9.509	-0.081	0.384	-9.206
23	c1ccc(cc1OC)O	9.65	15.103	5.453	13.169	3.519	10.977	1.327	9.242	-0.408	1.720	-7.930
24	c1cc(ccc1O)CO	9.82	15.006	5.186	13.407	3.587	11.382	1.562	9.496	-0.324	1.848	-7.972
25	c1cc(cc(c1)O)CO	9.83	15.149	5.319	13.400	3.570	10.939	1.109	9.277	-0.553	1.476	-8.354
26	c1cc(cc(c1)O)CC	9.90	15.668	5.768	13.909	4.009	11.730	1.830	9.848	-0.052	0.970	-8.930
27	c1cc(c(c1)O)CO	9.92	13.256	3.336	13.192	3.272	10.876	0.956	9.231	-0.689	1.705	-8.215
28	c1ccc(c(c1)c1ccccc1)O	9.93	15.207	5.277	13.503	3.573	11.573	1.643	9.869	-0.061	0.485	-9.445
29	c1ccc(c(c1)OC)O	9.93	15.629	5.699	13.288	3.358	11.179	1.249	8.878	-1.052	2.149	-7.781
30	c1ccc(cc1C(=O)[O])O	9.94	15.985	6.045	14.187	4.247	11.909	1.969	9.747	-0.193	1.593	-8.347
31	c1(ccc(cc1)O)F	9.95	15.276	5.326	13.498	3.548	10.971	1.021	9.556	-0.394	1.213	-8.737
32	c1(ccc(cc1)O)O	9.96	16.398	6.438	14.705	4.745	12.747	2.787	10.610	0.650	2.391	-7.569
33	c1ccc(cc1)O	9.98	15.444	5.464	13.553	3.573	11.260	1.280	9.601	-0.379	1.210	-8.770
34	c1cc(ccc1O)CC	10.00	15.965	5.965	14.214	4.214	12.030	2.030	10.414	0.414	0.632	-9.368
35	c1ccc(cc1C)O	10.08	15.693	5.613	13.906	3.826	11.476	1.396	10.102	0.022	1.650	-8.430
36	c1(ccc(cc1)O)C	10.19	16.015	5.825	14.441	4.251	12.162	1.972	10.292	0.102	1.347	-8.843
37	c1(ccc(cc1)O)OC	10.20	16.298	6.098	14.752	4.552	12.361	2.161	10.453	0.253	1.804	-8.396
38	c1cc(c(cc1)O)CC	10.20	15.824	5.624	14.024	3.824	11.874	1.674	9.924	-0.276	1.775	-8.425
39	c1ccc(c(c1)C)O	10.28	15.613	5.333	13.875	3.595	11.566	1.286	9.809	-0.471	1.760	-8.520
	MAE			4.901		3.467		1.379		0.463		7.504
	MSE			5.036		3.529		1.534		0.602		7.614
	MaxAbsError			7.407		4.790		3.718		1.410		9.477

Table S13: Predictions on anilines from the Thapa & Raghavachari **Set-I** dataset.[1]

			SMD only		SMD + 1 water		SMD + 2 waters		SMD + 3 waters		QupKake	
S.N.	SMILES	pK_a^{Exp}	pK_a	ΔpK_a	pK_a	ΔpK_a	pK_a	ΔpK_a	pK_a	ΔpK_a	pK_a	ΔpK_a
1	c1ccc(c(c1)N(=O)=O)N	0.28	-4.551	-4.831	-2.201	-2.481	-1.370	-1.650	-1.373	-1.653	1.242	0.962
2	c1(ccc(cc1)N)N(=O)=O	0.98	-2.883	-3.863	-1.253	-2.233	0.137	-0.843	1.220	0.240	2.499	1.519
3	c1ccc(c(c1)C(=O)O)N	2.04	-0.664	-2.704	0.480	-1.560	1.671	-0.369	1.671	-0.369	2.620	0.580
4	c1(ccccc1N)C(=O)OCC	2.10	-0.023	-2.123	0.660	-1.440	1.975	-0.125	1.975	-0.125	2.945	0.845
5	c1(ccccc1N)C(=O)OC	2.16	-0.150	-2.310	0.436	-1.724	1.770	-0.390	1.793	-0.367	3.015	0.855
6	c1(ccc(cc1)N)C(=O)OC	2.30	-0.602	-2.902	0.561	-1.739	1.582	-0.718	2.388	0.088	2.892	0.592
7	c1(ccc(cc1)N)C(=O)O	2.32	-1.164	-3.484	0.006	-2.314	1.540	-0.780	2.722	0.402	2.666	0.346
8	c1(ccc(cc1)N)C(=O)OCC	2.38	-0.747	-3.127	0.407	-1.973	1.394	-0.986	2.545	0.165	2.896	0.516
9	c1ccc(cc1N(=O)=O)N	2.45	-1.118	-3.568	-0.115	-2.565	0.851	-1.599	2.013	-0.437	2.383	-0.067
10	Clc1ccccc1N	2.62	-1.305	-3.925	-0.450	-3.070	1.090	-1.530	3.215	0.595	2.966	0.346
11	c1ccc(c(c1)F)N	2.96	-0.541	-3.501	0.011	-2.949	1.359	-1.601	2.957	-0.003	3.084	0.124
12	c1ccc(cc1C(=O)O)N	3.05	0.209	-2.841	1.032	-2.018	2.107	-0.943	3.294	0.244	3.621	0.571
13	c1ccc(cc1Cl)N	3.32	0.232	-3.088	1.210	-2.110	2.635	-0.685	3.640	0.320	3.726	0.406
14	c1ccc(cc1F)N	3.38	0.620	-2.760	1.485	-1.895	2.313	-1.067	3.257	-0.123	3.396	0.016
15	c1(cc(ccc1)N)C(=O)OC	3.56	0.566	-2.994	1.228	-2.332	2.132	-1.428	3.211	-0.349	3.914	0.354
16	c1ccc(c(c1)c1ccccc1)N	3.78	1.126	-2.654	1.557	-2.223	3.198	-0.582	5.029	1.249	3.746	-0.034
17	c1(ccc(cc1)N)Cl	3.81	0.796	-3.014	1.713	-2.097	3.144	-0.666	3.951	0.141	3.867	0.057
18	c1(cccc(c1)N)SC	4.05	1.487	-2.563	1.719	-2.331	3.328	-0.722	4.251	0.201	4.123	0.073
19	c1ccc(cc1O)N	4.17	1.592	-2.578	2.172	-1.998	3.502	-0.668	4.042	-0.128	3.697	-0.473
20	c1(cc(ccc1)N)OCC	4.17	1.722	-2.448	2.339	-1.831	3.187	-0.983	3.858	-0.312	4.056	-0.114
21	c1ccc(cc1OC)N	4.20	1.473	-2.727	2.344	-1.856	3.560	-0.640	4.271	0.071	4.062	-0.138
23	c1ccc(c(c1)C)N	4.38	2.168	-2.212	2.455	-1.925	4.150	-0.230	4.522	0.142	4.533	0.153
24	c1(ccc(cc1)N)SC	4.40	2.101	-2.299	2.820	-1.580	3.730	-0.670	5.081	0.681	4.434	0.034
25	c1(c(cccc1)N)OCC	4.47	1.762	-2.708	1.926	-2.544	2.829	-1.641	5.108	0.638	3.873	-0.597
26	c1ccc(c(c1)OC)N	4.49	1.747	-2.743	1.688	-2.802	2.746	-1.744	4.418	-0.072	3.635	-0.855
27	c1(ccc(cc1)N)F	4.52	1.542	-2.978	2.416	-2.104	3.413	-1.107	4.559	0.039	3.593	-0.927
29	c1ccc(cc1C)N	4.67	2.080	-2.590	2.736	-1.934	3.883	-0.787	4.700	0.030	4.643	-0.027
30	c1ccc(c(c1)O)N	4.72	0.075	-4.645	1.924	-2.796	3.276	-1.444	4.223	-0.497	3.600	-1.120
31	c1(ccc(cc1)N)C	5.07	2.459	-2.611	2.920	-2.150	4.198	-0.872	5.317	0.247	4.603	-0.467
32	c1(ccc(cc1)N)OCC	5.25	2.745	-2.505	3.138	-2.112	4.266	-0.984	5.557	0.307	5.304	0.054
33	c1(ccc(cc1)N)OC	5.29	2.824	-2.466	3.344	-1.946	4.430	-0.860	5.655	0.365	5.138	-0.152
34	c1(ccc(cc1)N)O	5.50	2.759	-2.741	3.311	-2.189	4.002	-1.498	5.176	-0.324	4.872	-0.628
	MAE			2.953		2.151		0.963		0.341		0.438
	MSE			3.021		2.186		1.058		0.481		0.577
	MaxAbsError			4.831		3.070		1.744		1.653		1.519

Table S14: Predictions on benzoic acids from the Thapa & Raghavachari **Set-I** dataset.[1]

			SMD only		SMD + 1 water		SMD + 2 waters		SMD + 3 waters		QupKake	
S.N.	SMILES	pK_a^{Exp}	pK_a	ΔpK_a	pK_a	ΔpK_a	pK_a	ΔpK_a	pK_a	ΔpK_a	pK_a	ΔpK_a
1	c1ccc(c(c1)N(=O)=O)C(=O)O	2.17	2.475	0.305	3.435	1.265	3.242	1.072	2.374	0.204	2.104	-0.066
2	Clc1ccccc1C(=O)O	2.94	3.776	0.836	3.723	0.783	3.678	0.738	2.413	-0.527	2.199	-0.741
3	c1ccc(c(c1)C(=O)O)C(=O)O	2.95	4.576	1.626	4.360	1.410	4.411	1.461	3.399	0.449	3.842	0.892
4	c1ccc(c(c1)O)C(=O)O	2.98	5.421	2.441	2.454	-0.526	2.135	-0.845	3.954	0.974	2.799	-0.181
5	c1ccc(c(c1)F)C(=O)O	3.27	4.878	1.608	2.519	-0.751	4.528	1.258	3.488	0.218	1.932	-1.338
6	c1ccc(cc1N(=O)=O)C(=O)O	3.45	4.599	1.149	4.763	1.313	4.290	0.840	3.483	0.033	2.461	-0.989
7	c1(c(cccc1)C(=O)O)C(C)(C)C	3.46	5.732	2.272	5.289	1.829	4.743	1.283	3.468	0.008	2.490	-0.970
8	c1(ccc(cc1)C(=O)O)C(=O)O	3.51	5.364	1.854	5.034	1.524	5.390	1.880	3.779	0.269	3.704	0.194
9	c1c(cc(cc1)C(=O)O)C(=O)O	3.54	5.374	1.834	5.441	1.901	5.537	1.997	3.692	0.152	3.623	0.083
10	c1(ccccc1C(=O)O)CC	3.77	5.283	1.513	4.960	1.190	4.829	1.059	3.589	-0.181	2.661	-1.109
11	c1ccc(cc1Cl)C(=O)O	3.83	5.289	1.459	5.295	1.465	4.758	0.928	3.661	-0.169	1.913	-1.917
12	c1ccc(cc1F)C(=O)O	3.87	5.409	1.539	5.385	1.515	4.838	0.968	3.711	-0.159	1.537	-2.333
13	c1ccc(c(c1)C)C(=O)O	3.91	5.435	1.525	5.211	1.301	4.945	1.035	4.026	0.116	2.390	-1.520
14	c1(ccc(cc1)C(=O)O)Cl	3.99	5.609	1.619	5.602	1.612	5.077	1.087	4.239	0.249	1.820	-2.170
15	c1ccc(cc1O)C(=O)O	4.08	6.161	2.081	6.117	2.037	5.576	1.496	4.527	0.447	2.649	-1.431
16	c1ccc(c(c1)OC)C(=O)O	4.09	5.384	1.294	6.427	2.337	4.749	0.659	4.071	-0.019	2.139	-1.951
17	c1cc(cc(c1)OC)C(=O)O	4.09	6.224	2.134	6.155	2.065	5.518	1.428	4.365	0.275	2.419	-1.671
18	c1(ccc(cc1)C(=O)O)F	4.14	5.915	1.775	5.820	1.680	5.304	1.164	4.355	0.215	2.142	-1.998
19	c1(cccc(c1)C(=O)O)OCC	4.17	6.159	1.989	5.811	1.641	5.595	1.425	4.967	0.797	2.348	-1.822
20	c1(ccccc1C(=O)O)OCC	4.21	5.388	1.178	5.127	0.917	5.104	0.894	3.863	-0.347	2.123	-2.087
21	c1c(cc(cc1)C(=O)O)C	4.24	6.181	1.941	6.003	1.763	5.692	1.452	4.743	0.503	2.257	-1.983
22	c1(ccc(cc1)C(=O)O)C	4.34	6.406	2.066	5.786	1.446	5.569	1.229	4.816	0.476	2.299	-2.041
23	c1(ccc(cc1)C(=O)O)C(C)C	4.35	6.350	2.000	6.062	1.712	5.726	1.376	4.977	0.627	2.338	-2.012
24	c1(ccc(cc1)C(=O)O)CC	4.35	5.801	1.451	5.564	1.214	5.061	0.711	4.228	-0.122	2.350	-2.000
25	c1(ccc(cc1)C(=O)O)OCC	4.45	6.632	2.182	6.275	1.825	5.863	1.413	4.648	0.198	2.828	-1.622
26	c1c(ccc(c1)OC)C(=O)O	4.47	6.535	2.065	6.349	1.879	5.792	1.322	4.809	0.339	2.986	-1.484
27	c1cc(ccc1O)C(=O)O	4.58	6.534	1.954	6.093	1.513	5.719	1.139	4.931	0.351	3.104	-1.476
28	c1(ccc(cc1)C(=O)O)N(=O)=O	4.92	4.672	-0.248	4.885	-0.035	4.351	-0.569	3.548	-1.372	2.467	-2.453
	MAE			1.941		1.445		1.169		0.350		1.448
	MSE			1.723		1.526		1.217		0.459		1.603
	MaxAbsError			2.441		2.337		1.997		1.372		2.453

Table S15: Predictions on carbon acids from the Thapa & Raghavachari **Set-I** dataset.[1] QupKake was not trained on carbon acids, which resulted in no predictions.

			SMD only		SMD + 1 water		SMD + 2 waters		SMD + 3 waters		QupKake	
S.N.	SMILES	pK_a^{Exp}	pK_a	ΔpK_a	pK_a	ΔpK_a	pK_a	ΔpK_a	pK_a	ΔpK_a	pK_a	ΔpK_a
1	c1ccc2c(c1)c1c([C@H]2C(=O)C)cccc1	9.9	13.848	3.948	12.932	3.032	11.461	1.561	9.817	-0.083	–	–
2	C(=O)[C@H](c1ccccc1)c1ccccc1	10.4	14.953	4.553	14.159	3.759	12.715	2.315	10.325	-0.074	–	–
3	c1ccc2c(c1)c1c([C@H]2C(=O)SC)cccc1	10.5	13.372	2.872	12.973	2.473	11.621	1.121	10.164	-0.336	–	–
4	c1ccc2c(c1)c1c([C@H]2C(=O)OC)cccc1	11.5	13.812	2.312	12.647	1.147	12.602	1.102	10.845	-0.665	–	–
5	C(=O)Cc1ccccc1	13.1	18.727	5.627	18.632	5.532	14.54	1.44	13.204	0.104	–	–
6	C(=O)(CNC(=O)C)c1ccc(cc1)C	14.8	20.645	5.845	18.196	3.396	–	–	15.005	0.205	–	–
7	C(=O)C	16.7	23.613	6.913	21.613	4.913	19.534	2.834	17.323	0.623	–	–
8	C(=O)(C)c1ccccc1	18.3	24.955	6.655	22.903	4.603	20.931	2.631	18.821	0.521	–	–
9	C(=O)(C)c1ccc(cc1)C	19.2	25.511	6.311	23.53	4.33	21.274	2.075	20.083	0.883	–	–
10	C(=O)(C)C	19.3	26.728	7.428	24.793	5.493	22.933	3.633	21.023	1.723	–	–
	MAE			5.250		3.870		2.079		0.521		–
	MSE			5.250		3.870		2.079		0.291		–
	MaxAbsError			7.430		5.530		3.633		1.723		–

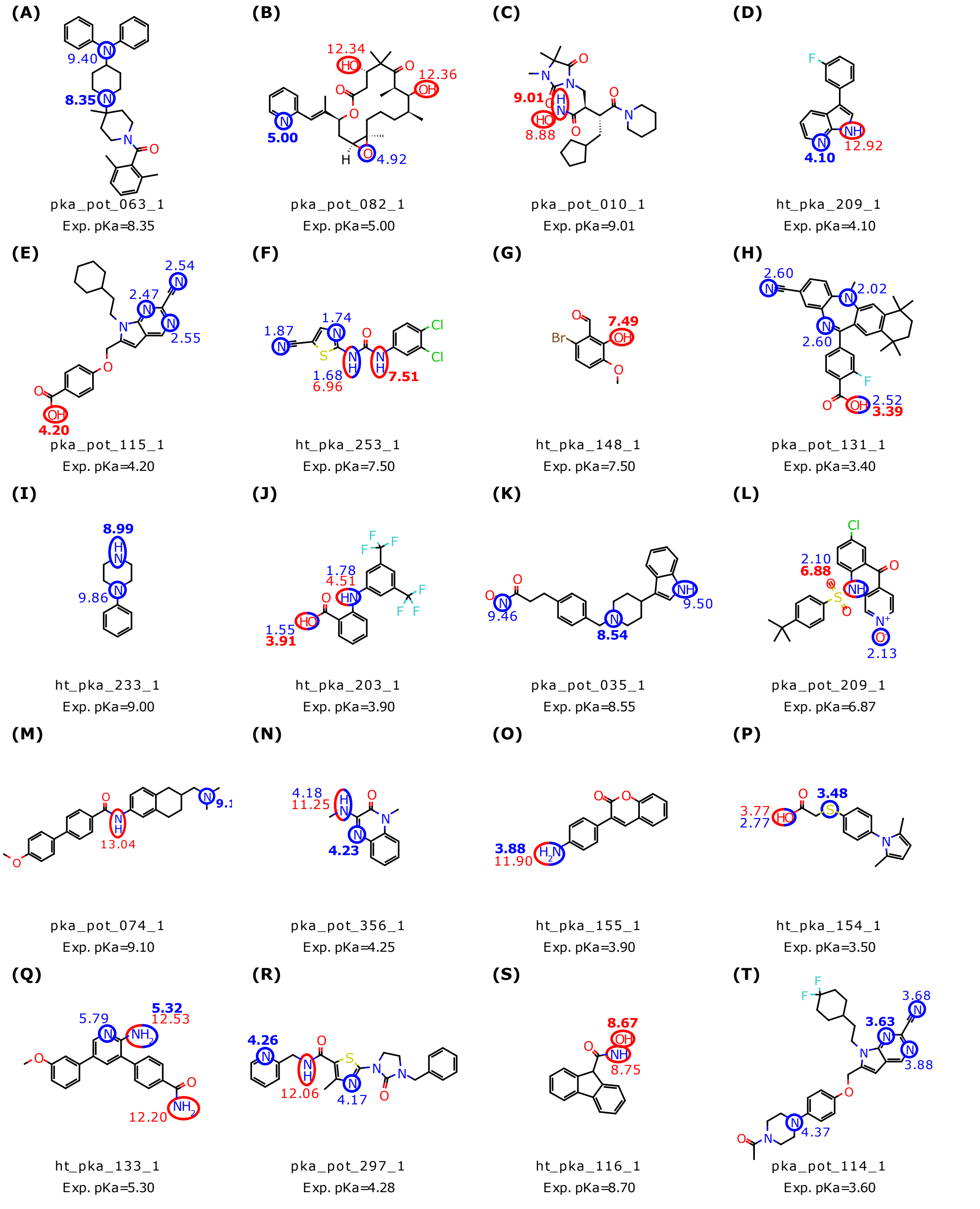

Figure S19: The top 20 molecules from the Novartis test set with the **most** accurate micro-pK_a prediction. The acidic and basic micro-pK_a values, as well as the atom they belongs to, are shown in red and blue, respectively. The micro-pK_a that is closest to the experimental value is shown in bold. The name, as it appear in the dataset, and the experimental pK_a value of each molecule are shown under the molecule.

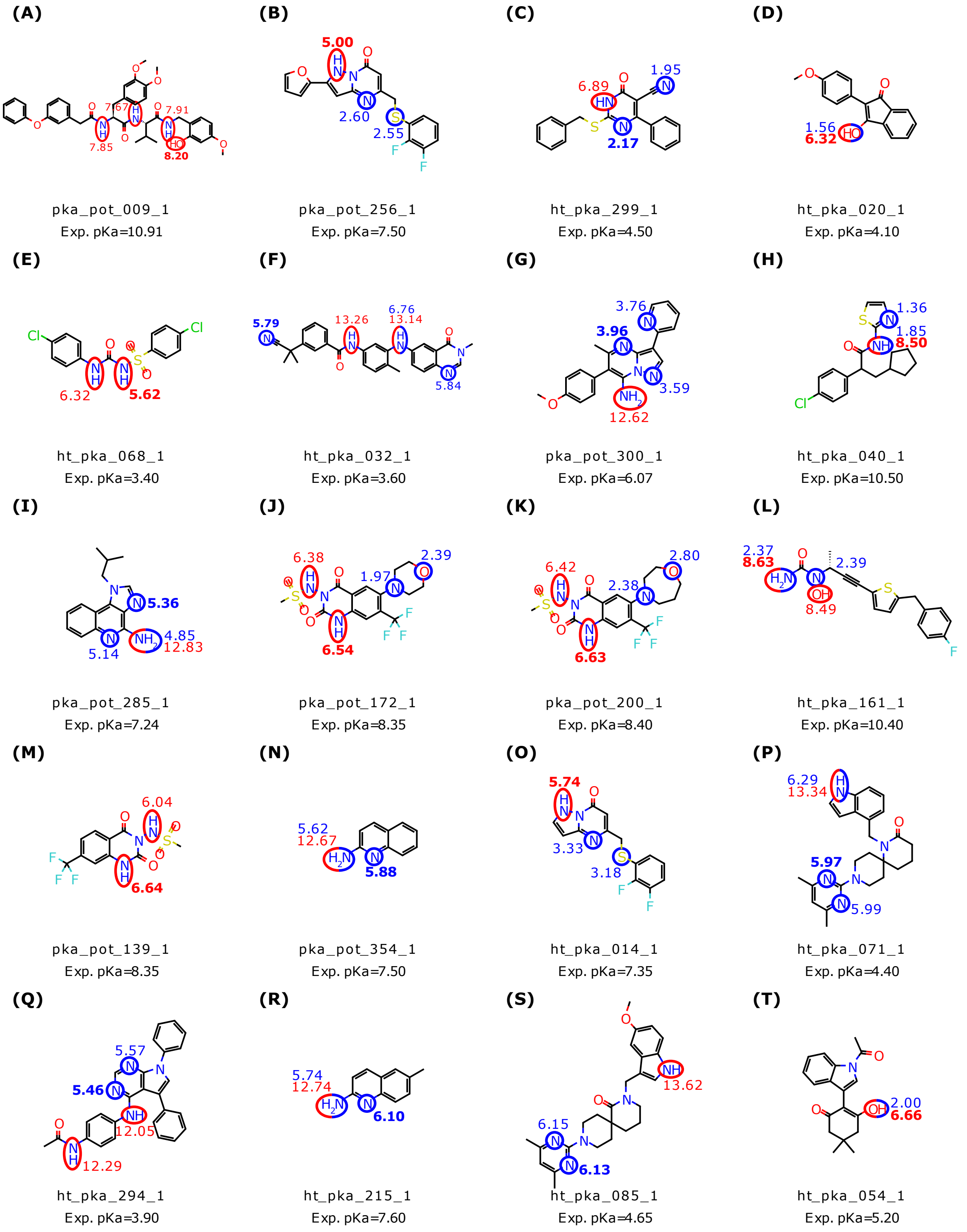

Figure S20: The bottom 20 molecules from the Novartis test set with the **least** accurate micro-pK_a prediction. The acidic and basic micro-pK_a values, as well as the atom they belongs to, are shown in red and blue, respectively. The micro-pK_a that is closest to the experimental value is shown in bold. The name, as it appear in the dataset, and the experimental pK_a value of each molecule are shown under the molecule.

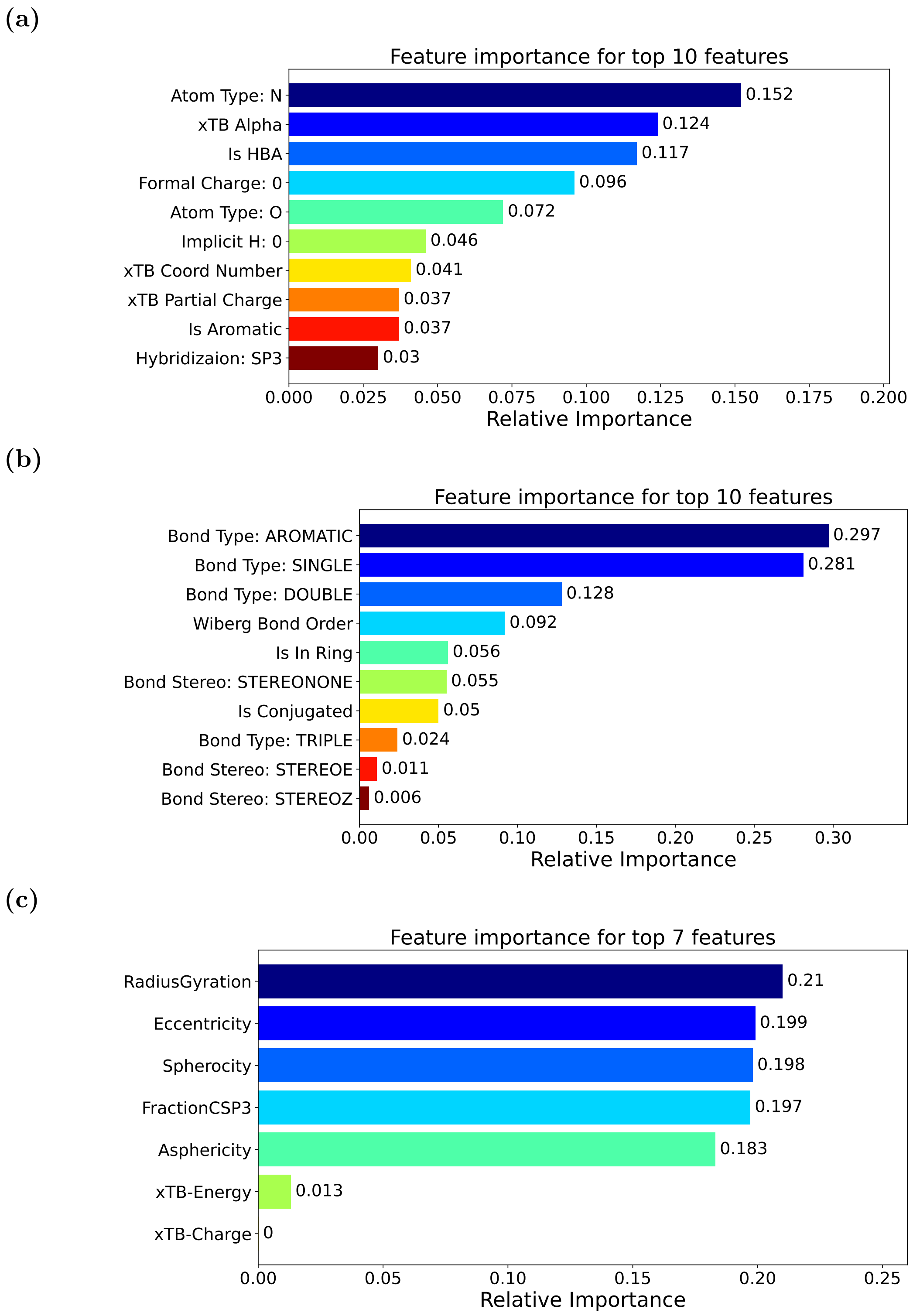

Figure S21: The normalized absolute relative importance of the (a) atomic features, (b) bond features and (c) molecular features for the micro-pK_a prediction model.

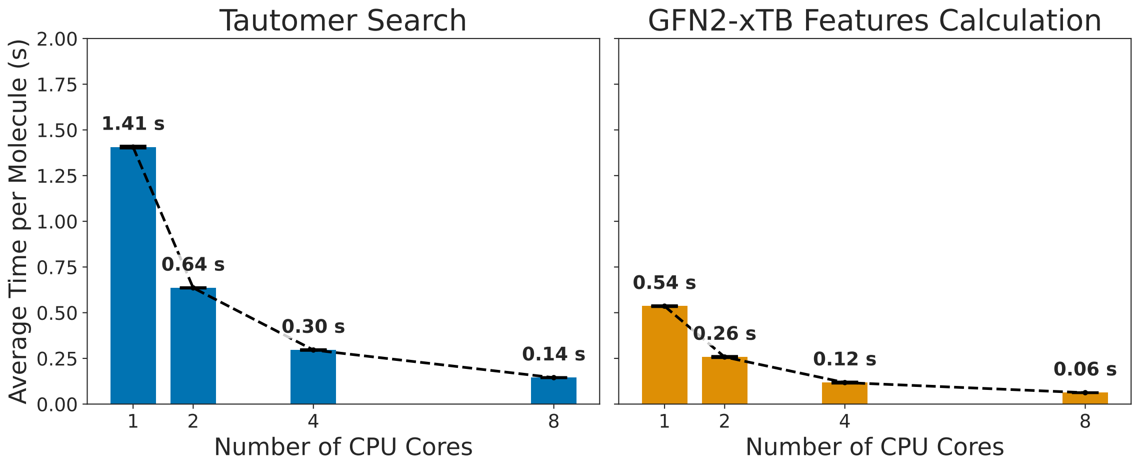

Figure S22: Tautomer search and GFN2-xTB features calculations average compute time per molecule across the 280 molecules in the Novartis test set as a function of the number of CPU cores.

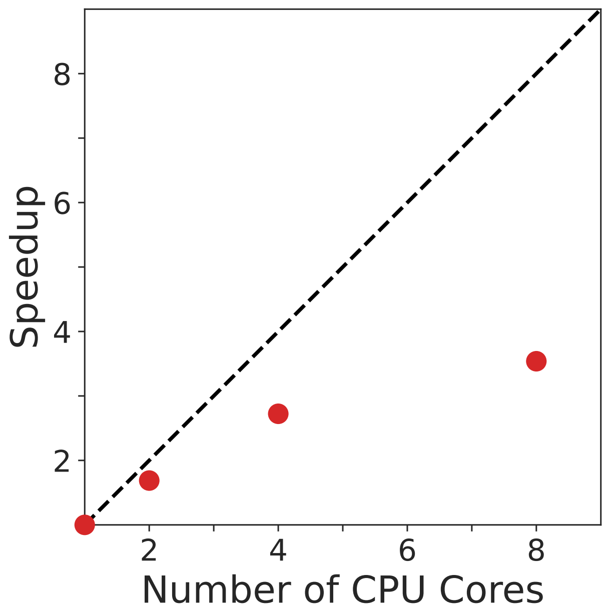

Figure S23: Compute time speedup rate across the 280 molecules in the Novartis test set as a function of CPU cores.

References

(1) Thapa, B.; Raghavachari, K. Accurate pKa Evaluations for Complex Bio-Organic Molecules in Aqueous Media. *Journal of Chemical Theory and Computation* **2019**, *15*, 6025–6035.
